# Screening for E3-Ubiquitin ligase inhibitors: challenges and opportunities

**DOI:** 10.18632/oncotarget.2431

**Published:** 2014-09-03

**Authors:** Vivien Landré, Barak Rotblat, Sonia Melino, Francesca Bernassola, Gerry Melino

**Affiliations:** ^1^ Medical Research Council, Toxicology Unit, Leicester, UK; ^2^ Biochemistry Laboratory, IDI-IRCCS, c/o Department of Experimental Medicine and Surgery, University of Rome “Tor Vergata”, Rome, Italy

**Keywords:** HECT, ITCH, p73, p63, high throughput screening, therapeutics, clomipramine, small molecular inhibitor

## Abstract

The ubiquitin proteasome system (UPS) plays a role in the regulation of most cellular pathways, and its deregulation has been implicated in a wide range of human pathologies that include cancer, neurodegenerative and immunological disorders and viral infections. Targeting the UPS by small molecular regulators thus provides an opportunity for the development of therapeutics for the treatment of several diseases. The proteasome inhibitor Bortezomib was approved for treatment of hematologic malignancies by the FDA in 2003, becoming the first drug targeting the ubiquitin proteasome system in the clinic. Development of drugs targeting specific components of the ubiquitin proteasome system, however, has lagged behind, mainly due to the complexity of the ubiquitination reaction and its outcomes. However, significant advances have been made in recent years in understanding the molecular nature of the ubiquitination system and the vast variety of cellular signals that it produces. Additionally, improvement of screening methods, both *in vitro* and in silico, have led to the discovery of a number of compounds targeting components of the ubiquitin proteasome system, and some of these have now entered clinical trials. Here, we discuss the current state of drug discovery targeting E3 ligases and the opportunities and challenges that it provides.

## INTRODUCTION

Protein ubiquitination followed by proteasomal proteolysis is the most common pathway of selective protein degradation in the cell [1-5]. However, in addition to signalling degradation, ubiquitination has been shown to be involved in the regulation of almost every cellular function [6]. In fact, up to 5% of the *Arabidopsis* genome encode genes that are part of the ubiquitin machinery illustrating the importance as well as the ubiquitousness of this post-translational modification [7]. It is therefore not surprising that deregulation of ubiquitin pathways has been implicated in the pathogenesis of numerous human disorders including cancer, neurodegeneration and inflammation [8-12].

**Figure 1:**
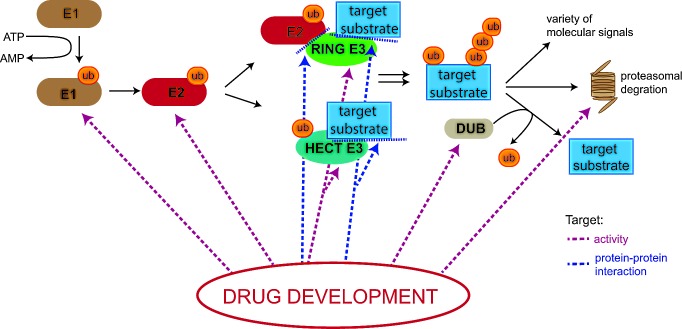
Potential drug targets in the Ubiquitin Proteasome System (UPS) Drugging the UPS has become a major research interest in recent years and several drugs targeting various components of the machinery are currently in clinical and pre-clinical development. Small molecules and peptides are being developed that either affect the intrinsic activity of enzymes involved in the cascade (depicted as red dotted lines) or interfere with protein-protein interactions (depicted as blue dotted lines). E1= ubiquitin- activating enzyme; E2= ubiquitin conjugating enzyme, ub=ubiquitin; ATP= adenosine triphosphate; AMP= adenosine monophosphate.

Targeting the ubiquitin proteasome system (UPS) by small molecule inhibitors would provide an appropriate way to regulate the levels and/or activity of single or sets of specific protein substrates, and thus an exciting opportunity for therapeutic interventions. Hence, since the discovery of the ubiquitin-proteasome pathway and especially after the clinical success of the proteasome inhibitor Bortezomib, targeting the UPS for therapeutics has become a research focus in academia as well as in pharmaceutical research [13]. However, identification of drugs that specifically target components of the ubiquitin cascade has lagged behind. In contrast, the field of kinase inhibitors accelerated after the approval of the first kinase inhibitor Gleevec in 2001, since a further 25 kinase inhibitors have been approved by the FDA for clinical use and many more are in clinical trials today [14, 15]. In 2003, Bortezomib was approved by the FDA for treatment of multiple myeloma, although no drug targeting other components of the UPS has been approved for clinical application since [16]. The clinical success of Bortezomib resulting from the complete block of proteasomal degradation came as a relative surprise as the UPS controls the levels of most cellular proteins. Indeed, its complete inhibition is expected to have disastrous effects on cellular homeostasis and exhibit cytotoxicity. Despite several theories, the mechanism by which this drug induces cell death in malignant relative to normal cells, is unclear, as well the reasons why it is proven a beneficial therapy in some cancer types but not others. Research efforts to identify compounds that target specific components of the UPS is underway, and aim at reducing the toxicity of the treatment, circumventing resistance and targeting a broader range of malignant diseases. One approach is to target components within the ubiquitination cascade to increase the specificity of the treatment to a subset of proteins or even to a single substrate. This approach would provide a much more elegant and expectantly less toxic strategy to specifically target cancer cells (Figure 1).

This review aims to provide an overview of the current state of drug discovery strategies involving the UPS, especially focusing on one class of E3 ubiquitin ligases (E3s), the HECT (Homologous to the E6-AP Carboxyl Terminus) enzymes, that so far have received little attention in the field of UPS related drug discovery.

### Functions of Ubiquitination

Protein ubiquitination is a dynamic, reversible and coordinated post-translational modification that most commonly provides a cellular tag for proteasomal degradation. However, depending on the protein ubiquitination state (mono-, multi- or poly-ubiquitination) and on the type of ubiquitin chain, an array of other functions of ubiquitination has become apparent in recent years and the diverse effects of this modification are emerging. The ubiquitin machinery consists of an enzyme cascade comprising three enzymes: in a first step, the ubiquitin- activating enzyme (E1) adenylates and thereby activates an ubiquitin molecule which is then transferred to the ubiquitin conjugating enzyme (E2) [17, 18]. This ubiquitin charged E2 now binds an E3 ligase which catalyses the transfer of the ubiquitin onto a lysine on the target substrate [19]. Depending on the class of E3 ligase, the ubiquitin is either directly transferred from the E2 onto the substrate, with the E3 merely functioning as a scaffold for the reaction (U-box and RING (Really Interesting New Gene) E3s), or ubiquitin is transferred onto a cysteine residue in the enzyme's catalytic centre (HECT E3s), and is then transferred onto the target substrate [20-25]. Ubiquitination is a dynamic process that is negatively regulated by deubiquitinases (DUBs). These enzymes catalyse the deconjugation of ubiquitin from substrates or ubiquitin chains, acting as important regulators of the ubiquitin machinery [26-28].

Proteins can be modified by one (monoubiquitination) or a chain of ubiquitin molecules (polyubiquitination). A ubiquitin chain can be formed via linkage of any of the seven lysine residues in ubiquitin (K6, K11, K27, K29, K33, K48 and K63) [29, 30] or less commonly through the N-terminal methionine of ubiquitin (M1, linear chain) [31, 32]. The complexity of the system is further enhanced by the existence of several different types of chains: (i) single linkage, (ii) mixed linkages with different linkages in one chain, (iii) branched linkages where one ubiquitin molecule in the chain is attached to two ubiquitin molecules and lastly (iiii) chains consisting of a mixture of ubiquitin and other ubiquitin-like molecules e.g. SUMO have been observed [33, 34]. Taken together this leads to almost endless possibilities of protein modification by ubiquitin and ubiquitin like modifiers [10].

### Proteolytic functions of ubiquitination

Generally, a chain of at least four ubiquitin molecules linked by either K11 or K48 to a protein substrate is both necessary and sufficient for recognition by the proteasomal 19S regulatory particle. Longer ubiquitin chains increase the affinity of the proteasome for the substrate and thus the probability of its degradation [29, 34]. Ubiquitin chains linked by either of the seven lysine residues in ubiquitin can associate with the proteasome *in vitro*, suggesting a potential role in proteolysis for all of them [35-37]. This raises the question why certain chains appear to lead to degradation more commonly than others. One possible explanation could lie in the deubiquitination activity of the proteasome towards different chain linkages. A study by Jacobson *et al*. [38] showed that while K63- and K48-linked polyubiqutin chains associate with the proteasome, the deubiquitinating rate of the linear K63 chains is around 6 fold faster than that of the more tightly packed K48 chains. K63 linked chains are therefore released from the proteasome upon complete de-ubiquitination faster than K48 chains, which reside longer and are thus more likely to be degraded [38, 39].

Different studies have demonstrated that *in vivo*, chains linked via K6, K11, K27, K29 and K48 are involved in protein degradation [35]. In addition to being a key player in proteasomal degradation, ubiquitination is also involved in autophagy and lysosomal degradation. Ubiquitin chains linked by K63 were shown to mediate lysosomal degradation of membrane proteins [40, 41]. Furthermore, monoubiquitination of receptor tyrosine kinases has been demonstrated to recruit members of the endocytic pathway resulting in endocytosis and lysosomal degradation of the kinase [42-46]. Lastly, ubiquitin is involved in autophagy signalling by binding to autophagy receptors like p62/SQSTM1 and NBR1 resulting in autophagic degradation of the substrate [47, 48].

### Proteolysis-dependent regulation of transcriptiona factors

One of the best-studied and characterised function of the UPS is the control of transcription factor (TF) activity by targeted proteasomal destruction [49]. The UPS is deeply involved in the NF-κB activation pathway [12] that critically regulates inflammatory responses and contributes to the pathogenesis of malignancies including multiple myeloma. NF-κB activation relies on the phosphorylation-induced proteasomal degradation of inhibitory proteins of κB family (IκB), which sequester NF-κB in the cytoplasm in unstimulated cells. Following cell stimulation, I*κ*B is rapidly ubiquitinated by the SCFβ^TrCP^ E3 complex, and degraded by the proteasome, unveiling a nuclear localization sequence on the NF-*κ*B proteins [50, 51]. These are then released from IκBs, and translocate to the nucleus to induce the transcription of target genes. Another well-described example of TF controlled by the UPS is the main regulator of the hypoxic response, HIF-1α (hypoxia inducible factor-1α). Under normoxic conditions, the protein is constitutively expressed, but instantly degraded by VHL (von Hippel-Lindau)-mediated ubiquitination [52]. The VHL E3 complex only recognises the prolyl-hydroxylated form of HIF1α. Under hypoxic conditions prolyl-hydroxylation of HIF-1α by PHD (prolyl hydroxylase domain) enzymes is inhibited, thus abolishing VHL-mediated ubiquitination of HIF-1α and resulting in its stabilisation and accumulation [53, 54]. The transcription factor then transactivates the expression of several genes including GLUT1, VEGF and erythropoietin [52].

Another example of TFs regulated through the UPS are the transcription factors of the p53 family, p53, p63 and p73. All three TFs are regulated by posttranscriptional modifications including ubiquitination that signals for their degradation. While ubiquitination and subsequent degradation of the tumour suppressor p53 has been linked to a plethora of E3s, the E3 MDM2 (mouse double minute 2) is the main and the best characterised regulator of its protein levels and activity [55-58]. The other two family members, namely p63 and p73, share some of the tumour suppressive functions of p53 and, additionally, were shown to be involved in skin and neuronal development and stemness, respectively [59-62]. Both proteins are regulated at the level of transcription and degradation. The HECT E3 Itch (itchy E3 ubiquitin protein ligase) has been shown to be a primary regulator of both proteins by targeting them for ubiquitination-mediated degradation. Similarly to p53 and HIF-1α, the steady-state levels of p63 and p73 are kept low by constant ubiquitin-dependent degradation in unstressed cells [59, 63-69]. In response to DNA damage, Itch activity is attenuated by means of different mechanisms [70-72] and its inhibition results in p63/p73 stabilisation and activation. p73 and the ΔN isoform of p63 are regulated by the tumour suppressor WWOX, which binds both proteins, and antagonizes the Itch-ΔNp63 interaction, thereby stabilising ΔNp63 protein levels [73, 74]. The WW-domain containing protein, Pin1 was shown to interact with p63 and protect it from ubiquitination by the WW domain containing E3 ubiquitin protein ligase 1 (WWP1) [75, 76]. Itch is not the only E3 ligase regulating p73 stability. Indeed, p73 was additionally shown to be a substrate of the E3s Pin2 [77], SCF^FBXO45^ [78] and more recently of TRIM32 [79].

### Non proteolytic functions of ubiquitination

The relevance of ubiquitination has also been recognised in controlling other cellular functions including protein-protein interactions, subcellular localisation, transcription, epigenetic modifications, and DNA repair. Cell signalling processes are predominantly regulated by the K63-linked type of polyubiquitination [80]. However, other non-degradable types of polyubiquitin chains such K11 were described.

### Protein-protein interactions

In addition to being bound by the proteasome, ubiquitin and ubiquitin chains can interact with a range of different proteins that contain an ubiquitin-binding domain (UBD). Around 200 cellular proteins are estimated to contain a UBD with which they can recognise ubiquitinated proteins and act as effector molecules that signal to downstream cellular pathways [81]. Thus ubiquitin can act as ‘molecular glue’ connecting different proteins and aid complex formation. Conversely ubiquitin can mask a protein-binding site and inhibit binding of a certain subset of binding partners [82]. The oncogene GTPase, K-Ras, for example is activated by monoubiquitination at Lys-147. While ubiquitination of K-Ras has no effect on its ability to interact with and hydrolyse GTP or activate downstream targets, it severely abrogates interactions with factors involved in K-Ras deactivation [GTPase-activating proteins (GAPs)] by occupying the binding interface, hence leading to K-Ras activation [83, 84].

Additionally, protein ubiquitination can lead to conformational changes in the substrate, eventually inhibiting binding of other proteins or affecting protein activity. Ubiquitination of type 2 iodothyronine deiodinase (D2), an endoplasmic reticulum-resident type 1 integral membrane enzyme, leads to transient conformational changes within the D2-D2 dimer that inactivate the enzyme. Deubiquitination of the dimer leads to reactivation of its enzymatic activity, allowing tight regulation of its function [85].

### Cellular localisation

Several studies have linked ubiquitination with cytoplasmic localisation of proteins. For example, nuclear localisation of CTP-phosphocholine cytidyltransferase (CCT-) is blocked when it becomes ubiquitinated at a site that is close to its NLS; this prevents its interaction with importin-α that delivers proteins to the nucleus [86]. Ubiquitination can also promote cytosolic localisation by inducing nuclear export. For instance MDM2-catalysed monoubiquitination of p53 induces nuclear-cytosolic shuffling [87]. Export of p53 to the cytoplasm represses its transcription function and concomitantly fosters transcriptionally independent activities of p53, such as apoptosis induction and autophagy inhibition (Reviewed in [88]). Similarly, WWOX-mediated ubiquitination of p73 and p63 promotes their cytoplasmic localisation and subsequent transcriptional inactivation [73, 74, 89]. Furthermore, polyubiquitination of the regulatory SMAD, SMAD3 that plays an important role in the TGF-beta signalling pathway, results in its nuclear export and subsequent proteasomal degradation in the cytoplasm [90]. Less frequently, ubiquitination has been identified as signalling for nuclear import. An example is site-specific ubiquitination of the tumour suppressor phosphatase and tensin homolog on chromosome 10 (PTEN) that was shown to result in nuclear import and thus activation of the protein's transcriptional activity [91].

### Regulation of transcription factors by ubiquitin

Promoting degradation is only one of several mechanisms by which ubiquitination can control TF activity. Several recent studies have highlighted non-proteolytic outcomes of protein ubiquitination in the control of TF function. These range from changes in protein localisation by mono-ubiquitination to an intriguing direct link of TF activity and ubiquitination [92]. Indeed, ubiquitination of TFs provides some of the most striking demonstrations of the diverse consequences of protein ubiquitination. Not only does ubiquitination play a critical role in the negative regulation of transcription factors, by targeting them for destruction by the proteasome, there are also examples where ubiquitination of TFs promotes their activity. This model arises from the observations that in many cases there is a structural overlap between transactivation (TA) domains, the domains that are required for transcription factor activity and degrons, the domains that signal their destruction. This phenomenon is found in more than 30 transcription factors including the tumour suppressor proteins p53 and TAp63 discussed above [92].

One of the first studies linking TF activity and ubiquitination showed that the degron and TA domain of the transcriptional activator Myc are functionally connected and that ubiquitination of Myc is required for transcriptional activation [93, 94]. Subsequently, several other transcription factors were shown to require activity of their respective E3s in order to be fully active. These include HPV E2, Gal-4, FOXO4, NF-κB and Tat [95-99], although how ubiquitination leads to increased transactivation of these proteins remains elusive. Different mechanisms have been proposed, including recruitment of co-factors and/or parts of the proteasome to the site of transcription. An example is the viral transactivator Tat (HIV-1). The transactivation activity of Tat requires ubiquitination by the E3 ligase MDM2, which is believed to lead to recruitment of the 19S particle of the proteasome to the HIV-1 promoter activating Tat mediated transcription [96].

The fact that a number of transcription factors are both activated and degraded by ubiquitination and that several transcription factors have been shown to be most active when least stable has led to the development of the ‘licensing’ or ‘kamikaze model’ where monoubiquitination is required for the activation of TFs, but also inevitably leads to polyubiquitination and destruction of the protein [100, 101].

Taken together, there appears to be a strong connection between ubiquitination and transcription factor function. Because TFs are key proteins, each regulating the expression of a distinct set of genes in a context dependent manner, manipulation of their ubiquitination may be expected to have significant physiological consequences.

### Therapeutic targeting of the UPS

Due to the enormous potential for intervention on multiple pathologies, including cancer, the UPS represents a suitable pharmacological target for drug development. The discovery that proteasomal inhibition is a potent tool to selectively induce cell death in cancer cells compared with normal cells has resulted in the UPS becoming the focus of attention as a drug target in cancer biology. Selective cytotoxicity of Bortezomib towards cancer cells has been ascribed to their fast cell growth, which would impose a greater workload on the proteasome. The remarkable activity of the proteasome inhibitor Bortezomib in clinical trials led to its approval by the FDA in 2003 for treatment of relapsed or refractory multiple myeloma [16], and the drug was later approved in clinical trials for relapsed mantle cell lymphoma, and diffuse large B-cell lymphoma [102-104]. Bortezomib inhibits the chymotrypsin-like activity of the proteasome by reversible binding to the β5 subunit of the 20S proteasome thereby impeding all proteasomal activity and leading to accumulation of polyubiquitinated proteins in the cell [105, 106]. Several mechanisms have been proposed to explain how proteasome inhibition leads to cell death of cancer cells, including stabilization of the pro-apoptotic proteins p27KIP1, p53 and Bax, defective nuclear factor-kB activation, decreased interleukin-6 signalling and induction of oxidative and endoplasmic reticulum stress [107, 108]. Furthermore, it has been suggested that Bortezomib can inhibit angiogenesis [109] and additionally that depletion of the cellular ubiquitin pool by proteasome inhibition induces cell death in cancer cells [110].

Despite its relative success in the clinic, the therapeutic window of bortezomib is relatively narrow, with toxic side effects ranging from peripheral neuropathy, myelosuppression to cardiotoxicity, due to the accumulation of misfolded proteins [111]. Additionally, there is a relatively high incidence of both intrinsic and acquired resistance to Bortezomib. This is believed to be mainly mediated by increased mRNA and protein expression of the β5-subunit of the proteasome, mutations in the subunit that inhibit binding of Bortezomib, constitutive activation of the NF-κB signaling pathway and upregulation of the endoplasmic reticulum chaperone protein GRP78 and P-glycoprotein, a multidrug resistance protein [112, 113]. The FDA approved a second proteasome inhibitor, Carfilzomib, in 2012 for treatment of multiple myeloma for patients resistant to Bortezomib [114]. Carfilzomib is an irreversible inhibitor, which appears to be more potent and selective than Bortezomib. However, the use of proteasome inhibitors as anti-cancer drugs in the clinic is currently limited to lymphomas and multiple myeloma with little success in treatment of other tumours. Hence, there is a major interest to identify drugs targeting the proteasome that can be used for the treatment of non-haematological tumours, as well as drugs with reduced toxicity profiles and refractory to the development of drug resistance.

One approach to target the UPS by limiting non-specific side effects and enhancing pharmacological effectiveness and potency is to block specific upstream components such as E1, E2, E3 and DUB enzymes (Figure 1) [115-122]. Furthermore, deregulation of the ubiquitin system has been shown to be involved in a plethora of non-malignant diseases, promoting the idea that targeting specific parts of the machinery might therefore not only produce anti-cancer drugs, but could be used for treatment of a range of diseases [9, 11].

### E1 inhibitors

Targeting the human ubiquitin activating enzyme (UBE1) and therefore the first step of the ubiquitination cascade has become a focus in drugging the proteasome system. Like proteasome inhibition, this would inhibit degradation of all proteins that are targeted for destruction by the UPS. However, it would additionally affect pathways in which ubiquitination plays a regulatory, non-proteolytic role. A number of compounds that inhibit UBE1 activity have been identified (see review [123]), the first cell-permeable one being PYR-41. This compound is an irreversible pyrazone derivative, which irreversibly binds to the active cysteine in UBE1 thus abrogating its catalytic activity [124]. On the contrary, it did not display inhibitory activity against other thiol-containing enzymes [124]. PYR-41 is thought to kill tumour cells by inhibiting cytokine-induced NF-κB activation, and promoting p53 accumulation, thus being more effective towards cancer cells bearing wild-type p53.

In addition to UBE1, there are 7 other E1 enzymes in the cell responsible for activation of other ubiquitin–like modifiers. To date inhibitors against two other E1 enzymes, the SUMO-1 activating enzyme (SAE) [125-127] and NEDD8 activating enzyme (NAE) have been developed [128]. NAE inhibition indirectly affects the UPS as NEDDylation is essential for the activation of the SCF (Skp, Cullin, F-box containing complex) Cullin-containing E3 complexes and thus represents a more specific approach than blocking UBE1. The small molecule NAE inhibitor MLN4924 is an adenosine sulfamate analogue that covalently binds the nucleotide-binding site of NAE. This results in the formation of a NEDD8-MLN4924 adduct that blocks the enzymatic activity of NAE, hence abrogating cullin NEDDylation and the SCF activity [128]. This is particularly relevant in cancer as several substrates of SCF play fundamental roles in cancer development, and their stabilisation upon SCF inhibition leads to cell cycle arrest, senescence and apoptosis. These biological effects are likely achieved through the accumulation of p27, NRF2, CDC25A, HIF1α and IκB. MLN4924 was also found to inhibit tumour angiogenesis [129]. This compound is currently being tested in phase I/II clinical trials for treatment of hematologic and nonhematologic malignancies [130].

### E2 inhibitors

E2 enzymes, which act as intermediates between the E1 and E3 proteins, have been shown to play a role in determination of the type of the polyubiquitin chain linkage. Since the E2 enzymes bind E1, E3 and ubiquitin, they could be targeted at different interaction surfaces. Because each E2 can associate and cooperate only with a specific set of E3s, in principle, the more promising and specific approach would be to block the E2-E3 association. Thus far these enzymes have received less attention as drug targets than other parts of the ubiquitin cascade. Among the few compounds developed, CC0651, is an allosteric inhibitor of human Cdc34, the E2, which cooperates with the SCF E3 complexes. CC0651 binding to Cdc34 leads to conformational changes in the enzyme that impinge on the discharge of ubiquitin to acceptor lysine residues. The therapeutic value of this compound relies on the protection of p27^KIP1^ from degradation through the abrogation of its ubiquitination by SCF^Skp2^ [131]. Because p27^KIP1^ is a cyclin dependent kinase (Cdk) inhibitor that negatively regulates cell cycle progression, it may prove to be an important target for cancer therapy. In addition, NSC697923, an inhibitor of the Ubc13–Uev1A E2 enzyme blocks the formation of the E2–Ub thio-ester conjugate. Since Ubc13–Uev1A catalyses the formation of K63-linked poly-Ub chains, this compound has inhibitory activity against NF-κB signalling activation, which ultimately leads to reduced proliferation and viability of cancer cells [132].

### E3 inhibitors

The E3 is the last enzyme in the ubiquitin cascade that is responsible for substrate specificity. So far approximately 600 E3 ligases have been identified. Each E3 ligase can bind and ubiquitinate a set of substrates and hence inhibition of a particular E3 is expected to affect only the pathways that are regulated by that enzyme [82, 133]. The selectivity of ubiquitination provided by the E3s may address, at least in part, the specificity issue highlighted above and it is in contrast to the case of proteasome or E1 inhibitors. Specific targeting of a limited set of substrates theoretically may lead to fewer toxic side effects and to a more suitable targeted therapy.

E3s can be divided into three families: RING, U-box- and HECT-containing E3s. Both RING and U-box do not possess intrinsic catalytic activity and act by simultaneously binding to E2, ubiquitin and substrate, merely providing a scaffold for the ubiquitination reaction. Targeting RING or U-box E3s thus requires development of allosteric or protein-protein inhibitors. In contrast, as HECT E3s have intrinsic enzymatic activity, their inhibition implies blocking the catalytic site. Despite considerable research efforts, the identification of E3 inhibitors has been limited, partly due to the fact that researchers have primarily focused on disrupting the enzyme/substrate interaction that is considered more difficult to target than a catalytic site. Hence, even though, in principle, HECT E3 represent a more easily and promising druggable target than RING enzymes, so far they have received less attention as potential cancer therapeutics. As a result the majority of the small molecule inhibitors reported so far target RING-finger type enzymes, and several are currently undergoing evaluation in clinical trials [134].

The search for E3 inhibitors has mainly focused on a handful of E3s that have been found to be involved in cancer development, with a vast amount of research effort focusing on the identification of inhibitors of MDM2, IAP and SCF E3s (see table 1 for an overview of E3 inhibitors). There are three main strategies employed to develop inhibitors for E3 ligases, (i) to directly inhibit their enzymatic activity, (ii) to target the substrate binding interface or (iii) to affect the expression of the protein by transcription or translation inhibition. One concern when targeting the enzymatic activity is the fact that numerous E3s possess auto-ubiquitination capacity that generally promotes their degradation [135]. As a result, inhibition of E3 activity often abrogates its autoubiquitination, as well as substrate directed activity, and thus stabilises the E3 protein as well as its substrates. A stabilised pool of E3 that still binds, but not ubiquitinates its substrates, could have an unfavourable effect on the substrate activity. Formation of E3:substrate complexes could, for example, impair binding of other proteins to the substrate. This issue warrants consideration for the consequence of blocking the E3 activity entirely. Selected E3 as novel anticancer targets will be discussed below.

**Table 1:** E3 ligase inhibitors and method of identification

Target	Compound	Screening method	Validation	Reference
***MDM2***	Nutlins MI-63 Mel 23 HL198 TDP521252&TDP665759	*In vitro* screen using Biacore's surface plasmon resonance technology (MDM2/p53 interaction) Structure based design In cell screen (E3 ligase activity) *In vitro* HTS screen for compound that inhibit MDM2 autoubiquitination (MDM2 immobilised) *In vitro* HTS screen for inhibitors of the p53-MDM2 interaction using a thermal denaturation assay	*In vitro*, in cells, *in vivo*, now tested in clinical trials phase I *In vitro*, in cells, *in vivo* In cells In cells In cells	[234] [235, 236] [221] [237] [238]
***Skp2***	#25 C1, C2, C16, C20 NSC689857 and NSC681152	Virtual Screen (Skp2/Skp1 binding) Virtual Screen (Skp2/p27 binding) *In vitro* alpha screen assay (Skp2/Cks1 binding)	*In vitro*, cells, and *in vivo* *In vitro* and in cells *In vitro*	[201] [203] [202]
***IAP (XIAP, cIAP1, cIAP2)*** ***XIAP, ML-IAP, cIAP1, cIAP2***	Compound 2 (SM-406) Compound 1 (GDC-0152)	Rationale in silico design (SMAC mimic, inhibits SMAC/substrate binding) Structure-based design and targeted compound library generation	*In vitro*, in cell, *in vivo* Currently in clinical trial I *In vitro*, in cell, *in vivo*, Currently in clinical trial I	[239] [240]
***Frataxin (substrate)***	Compound (+)-1l)	Structure based virtual screen (binding to E3 ligase)	In cells	[209]
***VHL***	No name	Rational design (binding to HIF1a)	*In vitro* binding	[215, 216]
***SCFCdc4***	SCF-12	*In vitro* fluorescent polarisation screen (SCF-substrate)	*In vitro*	[205]
***SCFMet30***	SMER3	Yeast based screen	*In vitro* and in yeast cells	[204]
***Itch***	Clomipramine	*In vitro* HTS screen for Itch autoubiquitination (ELISA based assay)	*In vitro*, in cells	[230]
***E6AP***	CM_11_-1	Screen of a natural product like macrocylic N-methyl-peptide library using a display approach	*In vitro*	[232]

### MDM2

**Figure 2:**
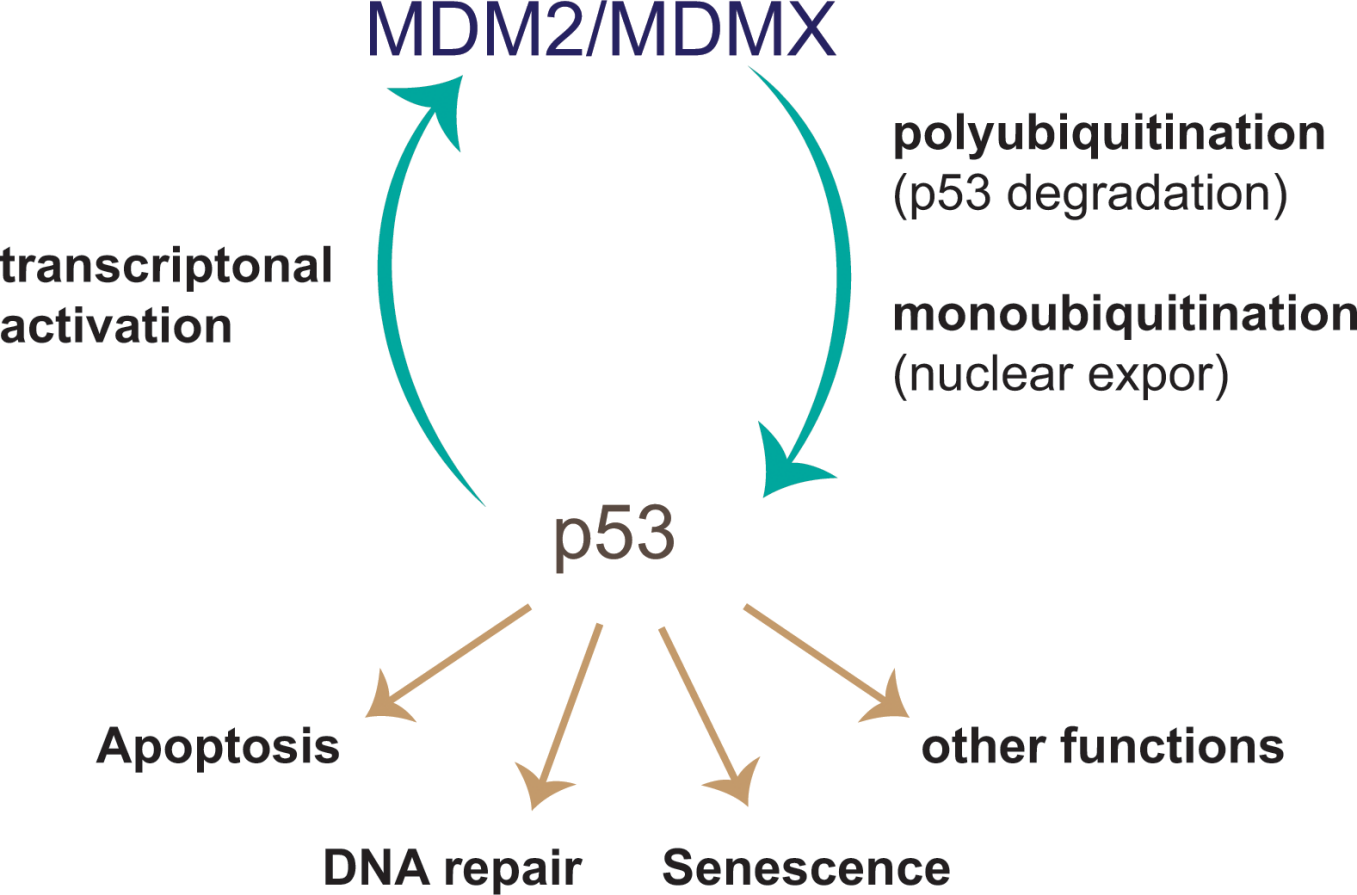
p53-MDM2 negative feedback loop In response to its activation the tumour suppressor p53 leads to expression of a number of target genes including its key regulator MDM2. The E3 ligase MDM2 then mediates p53 ubiquitination leading to its proteasomal degradation. This negative feedback ensures fine control of the duration of the p53 response and immediate termination upon loss of p53 stimulating signals. MDM2=Mouse double minute 2.

Since p53 inactivation is a crucial step in tumorigenesis, restoring its function is a hotspot for cancer drug development [136-138]. p53 is a transcription factor that has been shown to play a major role in the cell's response to oncogenic stresses and tumour suppression by inducing cell cycle arrest, senescence, anti-oxidative stress responses or apoptosis (reviewed in [139])[140]. With ~50% of all human malignancies carrying a mutation in the p53 gene, the tumour suppressor is one of the most commonly mutated proteins in human cancers [141]. The RING-type E3 MDM2 is the main regulator of the p53 tumour suppressor protein [55-57] (Figure 2). Under normal conditions, p53 levels are kept low by MDM2-mediated ubiquitination, leading to its degradation. Only in response to activating stimuli MDM2 is inhibited and p53 stabilised. Indeed, high activity of MDM2 has been observed in different cancers leading to the reduction or loss of p53 protein [142].

Targeting MDM2 by small molecules that prevent binding and/or ubiquitination of p53 has therefore been a major research interest in recent years and four compounds that block the MDM2-p53 interactions are currently being tested in phase I clinical trials (reviewed in [143]). The first inhibitor of the MDM2-p53 interaction was Nutlin. Nutlin was shown to bind to MDM2 and disrupt binding of the p53 N-terminus to the hydrophobic pocket of the E3; thereby abolishing MDM2 mediated suppression of p53. Subsequently, a number of compounds have been identified that disrupt the p53-MDM2 interaction and these are currently in pre-clinical development [143]. However, the first published results of phase I clinical trials with the second generation Nutlin compound, RG7112, for the treatment of liposarcoma have been rather disappointing. While analysis of patients biopsies showed an increase in p53 and p21 levels in response to RG7112 treatment, out of twenty patients only one showed a partial response, fourteen had stable disease and five showed disease progression. Furthermore, the drug showed relatively severe side effects including thromboycytopaenia and neutropaenia [144]. Results of two other clinical trials using the same compound in AML and soft tissue sarcoma are awaiting publication. The toxic side effects observed in the liposarcoma patients could be due to upregulation of PUMA and NOXA by stabilised p53 leading to apoptosis in normal cells, thus being the results of Nutlin on-target effects [143]. This illustrates one of the main concerns of p53 stabilising therapy, the effect of p53 activation on normal cells and whether cell death can be selectively induced in cancer over normal cells using p53 activators.

Results of the on-going clinical trials using different MDM2 inhibitors will hopefully shed light on the severity as well as prevalence of toxicity of MDM2 inhibitors, and might help to develop methods of administering the compounds in a less toxic manner. p53 can induce both cell cycle arrest and apoptosis and it was recently shown that the outcome of p53 activation is dependent on an expression threshold of p53 protein and its targets [145]. As cancer cells have been shown to be more sensitive to p53 induced apoptosis than normal cells, it has been suggested that administering MDM2 inhibitors in frequent small doses might circumvent apoptosis in healthy cells, but induce cell death in cancer cells [143]. More research into the effect of MDM2 inhibitors on healthy tissue will be necessary to fully exploit MDM2 inhibition in the clinic.

### ITCH

The members of the p53 family, p53, p63 and p73 have been shown to encompass overlapping target genes, interact with one another [146] and play similar biological roles [147-149]. These include common roles in the protection of the reproductive system [150-152], in development [153-159], aging [160, 161], cell death [162-164], cancer [159, 165-167], redox regulation [161, 168, 169], and metabolism [168, 170, 171]. Moreover, as is the case for p53 [172, 173], p63 is an important factor in cancer response to therapy as p63 and p53 are found in similar molecular complexes that mediate cisplatin resistance [174]. Furthermore, the ΔNp63 isoform was shown to mediate a pro-apoptotic response to cisplatin by regulating the expression of micro-RNAs [175]. Indeed, the ΔNp63 isoform directly induces the expression of the oncogenic mir-155, which drives tumor cell migration and growth [176] and p63 depletion promotes a metastasis program in prostate cancer through regulation of mir-205 [177]. p63 and p73 can activate an internal *p53* promoter and thereby control the expression of p53 isoforms [178], furthermore, p63 binds to mutant p53 affecting its tumorogenic functions [179, 180]. Structuraly, there are similarities between p53 and p73 as both are found in an ‘open’ tetrameric conformation while p63 is iterating between a ‘closed’ dimeric conformation under resting conditions and ‘open’ tetrameric conformation when activated by DNA damage [181].

**Figure 3:**
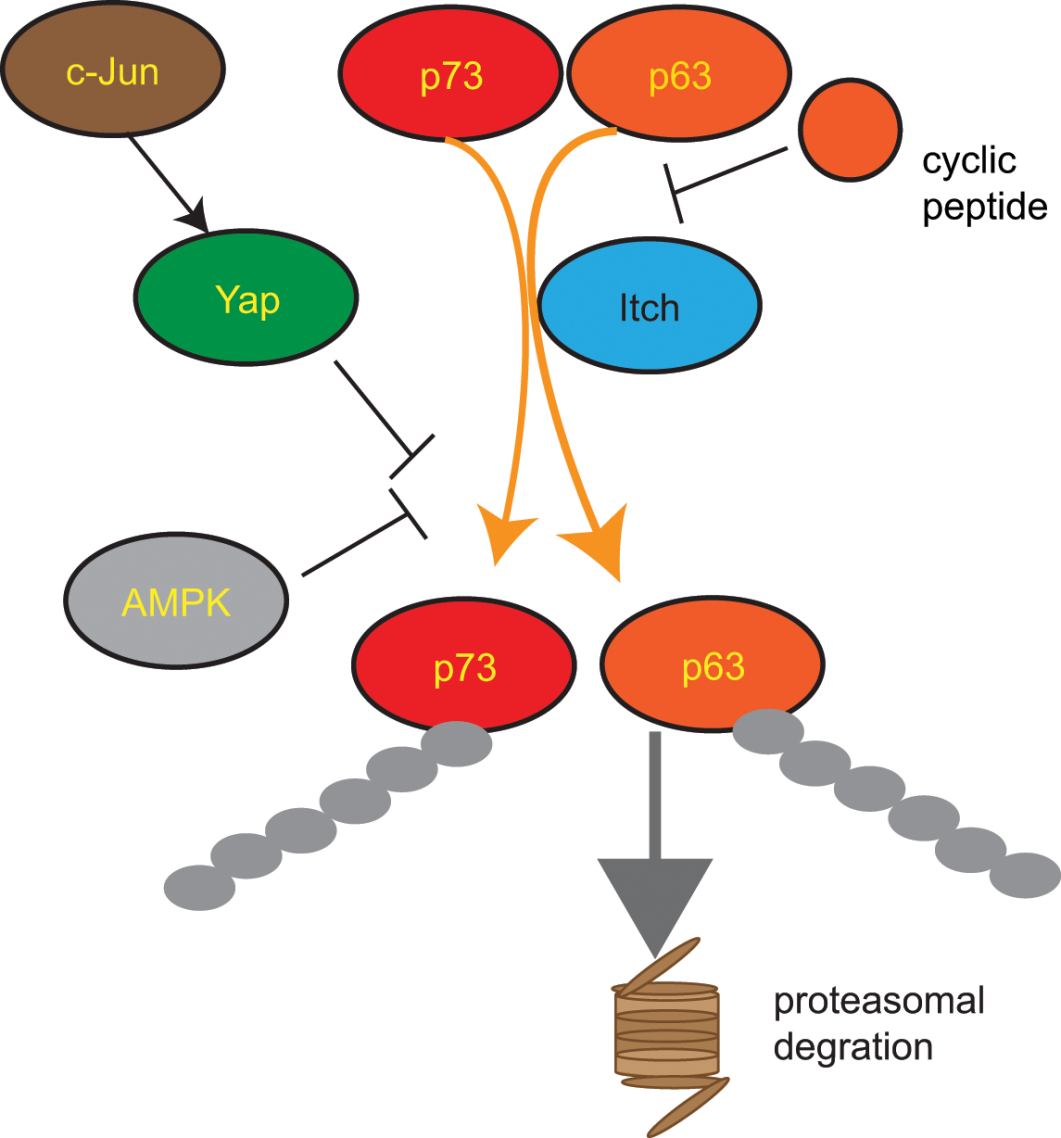
Regulation of the HECT E3 ligase Itch Itch is an important negative regulator of the transcription factors p63 and p73 leading to their polyubiquitination and subsequent proteasomal degradation p73 ubiquitination by Itch activity is inhibited by AMPK mediated phosphorylation of p73. c-Jun induces YAP expression which binds to Itch and inhibits its activity. Cyclic peptides mimicking the Itch binding interface on p63 inhibit Itch. AMPK= AMP-activated protein kinase; YAP= Yes-Associated Protein.

Blocking ubiquitination-mediated degradation of p63 and p73 is therefore a promising therapeutic strategy. While p53 is ubiquitinated by MDM2 [182], p63 and p73 are ubiquitinated by the HECT E3 Itch, which marks them for proteasomal degradation [67, 68]. Indeed, Itch inhibition results in increased sensitivity of cancer cells to cytotoxic drugs independently of their p53 status [89]. A possible strategy to inhibit p63 or p73 ubiquitination is to interfere with Itch binding. p63-Itch binding occurs through a PPxY motif found in p63 and the WW2 domain of Itch [68]. Furthermore, this interaction is facilitated by *tran/cis* proline isomerization of the adjacent *(T/S)P* motif by the Pin1 prolyl-isomerase [183]. In accord, a p63-PPxY motif containing cyclic peptide was shown to bind Itch *in vitro* [64]. Moreover, the cyclic peptide binds metal and the peptide-metal complex was demonstrated to induce oxidative damage to the WW2 domain of Itch suggesting that this could be a therapeutic strategy to interfere with Itch-p63 interaction and the resulting p63 degradation [64, 65] (Figure 3).

Similarly to the Itch- p63 interaction, the interaction of Itch with p73 occurs through a PPxY motif found in p73 and the WW2 domain of Itch [68]. Interestingly, Yes-Associated Protein (YAP) binds to p73 [184], preventing Itch-p73 interaction and leading to increased p73 levels and activity [185]. This YAP-p73 axis was shown to be important in activation of apoptosis by c-Jun following DNA damage as YAP is a target gene of the active c-Jun [186] (Figure 3). p73-Itch interaction is also negatively regulated by the AMP-activated protein kinase (AMPK) which phosphorylates p73 and increases its stability [187] (Figure 3).

Inhibitors of Apoptosis (IAPs) A promising group of E3s as drug targets in cancer is the IAP (anti-apoptotic proteins) family [188] that is augmenting the anti-apoptotic NF-kB pathway and inhibiting the pro-apoptotic apoptotic caspases and Smac proteins [59, 189]. The IAP proteins c-IAP1, c-IAP2, XIAP, ML-IAP and ILP-2 contain a RING domain and ubiquitinate several molecules involved in apoptosis signalling, and cancer [190] thus affecting their function or leading to their degradation (reviewed in [191]). One example is C-IAP1/2 mediated polyubiquitination of RIP1 (Receptor-interacting protein 1) [192]. RIP1 promotes cell survival by activating NF-kB and polyubiquitination of RIP1 by K63 linked chains is required for this activity. The ubiquitin-RIP1 complex serves as a scaffold for assembly of the IKKα-IKKβ-IKKγ signalling complexes. Thus inhibition of RIP1-mediated ubiquitination inhibits NF-kB activation [192, 193]. This has stimulated pharmaceutical companies to developed IAP inhibitors based on the IAP- Smac interaction. The drugs were designed to mimic Smac and are reported to induce IAP autoubiquitination resulting both in its degradation and activation of the TNF-pathway that induces cell death. IAP inhibition also suppresses proinflammatory cytokine production by TLR signalling and it has been suggested that IAP inhibitors therefore might be able to be used for the treatment of chronic inflammatory diseases [14, 194, 195].

### Skp2

Another attractive E3 that could be exploited for cancer therapy is Skp2 (S-phase kinase-associated protein 2), an F-box protein that forms part of the SCF complex. Skp2 targets the cell cycle inhibitor p27 for ubiquitination and degradation and was shown to possess oncogenic properties [196]. Remarkably, p27 is downregulated in numerous malignancies, which is believed to be due to excessive SCF-mediated ubiquitination [197-200]. Several compounds have been identified that inhibit Skp2 activity and are currently in pre-clinical development [201-203]. In addition, inhibitors of other components of the SCF complex have been developed [204, 205].

### DUBs

DUBs are the enzymes that remove ubiquitin from modified proteins or ubiquitin chains and, not surprisingly, have been shown to be involved in the regulation of almost all ubiquitin-dependent pathways. Approximately 80 DUBs have been described in man and many of them have been implicated in disease development, suggesting that they may also form a potential drug target [9, 26, 206]. While inhibiting DUBs that deubiquitinate specific proteins leads to stabilisation of their substrates, targeting proteasome associated DUBs results in proteasome inhibition.

DUBs can be divided into six sub-families, which vary in the degree of their chain and substrate specificity: ubiquitin-specific proteases (USPs), ovarian-tumor proteases (OTUs), Machado-Joseph disease protein domain proteases, ubiquitin carboxy-terminal hydrolases (UCHs) and the recently identified monocyte chemotactic protein-induced protein (MCPIP). All DUBs are cysteine proteases with the exception of JAMMs which are metalloproteases [206]. Due to their catalytic centre, DUBs are predicted to be drugable targets, and inhibiting DUBs could circumvent the challenge of developing activators of E3 ligase activity.

A number of inhibitors have been developed that either target specific enzymes or DUB subgroups. Recently, Ernst *et al* [207] reported DUB inhibitors that are based on ubiquitin variants, as DUBs bind to ubiquitin or polyubiquitin chains. Using a phage display approach the authors identified ubiquitin mutants with an increased affinity to specific DUBs compared to wild type ubiquitin and found that these act as competitive inhibitors of DUB activity. The ubiquitin variants lack the two N-terminal glycine residues and can thus not be utilised by the E1, E2, E3 cascade for protein ubiquitination. The study furthermore identified ubiquitin variants that display high specificity and binding affinity for the HECT E3 ligases Itch and E6AP and the Cdc34 E2 enzyme. Further study of these molecules will be necessary to show if these can be used as effective inhibitors of E3 and E2 ligase activity respectively [207].

### Screening methods for E3 ligase inhibitors

### In silico

In silico approaches that screen large drug libraries are becoming increasingly popular as a first step in drug discovery. This is due to docking programs that are able to predict the free binding energy of receptor proteins to millions of compounds from virtual drug libraries. These are becoming faster and more accurate with advances in computer processing power and this technology is becoming more widely accessible [208]. Furthermore, crystal structures of proteins, which are required for the screen, are more readily available. Often, computer-based screens are coupled to a second molecular or cellular *in vitro* screen of compounds that obtained a high score in silico. Using virtual screening Chan *et al* [201] recently discovered an inhibitor of the tumor promoting E3 ligase Skp2, ♯25, which exhibited anti-tumour activity in cells and animal models. Skp2 is involved in cell cycle progression, metabolism, metastasis and senescence and was shown to be deregulated in a number of cancers. Compound ♯25 was identified using a structure-based high-throughput virtual screen of 120,000 drugs linked to an *in vitro* binding assay of purified Skp2 protein of the top hits. Validation of compound ♯25 showed that it effectively inhibits Skp1/Skp2 interaction and Skp2 mediated p27 ubiquitination as well as tumour growth in a mouse xenograft model [201].

Another interesting approach to inhibit ubiquitination of a specific substrate is to block the ubiquitination of the target rather than blocking the E3 ligase and therefore the ubiquitination of all its targets. This was demonstrated by a study that identified an inhibitor of the ubiquitination of frataxin, a mitochondrial protein whose downregulation is linked to Friedreich's ataxia, a neuro-and cardiodegenerative disorder. Lavecchia *et al*. [209] identified the ubiquitination site on frataxin and modelled the position of ubiquitin on the protein surface using in silico docking programs. Next, they used structure based virtual screening coupled with a cell-based assay of the 13 top hits, and identified a small molecule that disrupts the frataxin-ubiquitin interactions and thereby inhibits frataxin ubiquitination [209].

In addition to using in silico screens as a first step to identify potential drug candidates from a drug library, computational methods have also been found useful in characterising and optimising existing E3 ligase inhibitors. The known crystal structure of protein complexes can be used to identify peptides within a protein-protein interface that can be used to inhibit the interaction and then optimised further using molecular modelling techniques [210]. This approach provides several advantages, as peptide inhibitors are expected to be more selective and potent due to intrinsic peptide flexibility that can adapt to protein surfaces [210]. Another advantage of using peptides as therapeutic agents is that they accumulate less in tissue. The main challenge of using peptides in a clinical scenario is due to their poor metabolic stability as well as cell membrane permeability. These limitations may be overcome by development of stapled peptides that can be used to stabilise alpha helical structures that renders the peptides resistant to proteolysis and increases their cell permeability. The technique, developed by Blackwell and Grubbs, [211], utilises non- natural amino acids to form an all-carbon cross link of the peptide. Several groups are currently developing stapled peptide inhibitors of MDM2 that bind to the p53-binding interface on MDM2 and thus abrogate the MDM2-p53 interactions [212-214]. Furthermore using in silico modelling, a known E3 ligase-substrate interaction site can be exploited as a template for rationale design of small molecular inhibitors. Buckeley *et al*. [215, 216] developed a hydroxyproline analogue inhibitor of the VHL (von Hippel-Lindau) E3 ligase, the main regulator of HIF-1α, based on the interaction between Hyp564 on HIF-1α and VHL.

### In vitro

*In vitro* HTS are based on identification of compounds that lead to drug-induced interference of E3-substrate interaction or reduction of substrate or E3 ubiquitination. The assays developed so far can be divided in two sub-groups. In the first type of assay all components are free in solution, whereas in the second, the substrate (target protein or E3 ligase) is immobilised on beads or a plate with the other components of the reaction in solution.

Several fluorescence-based assays that measure the proximity of either E3-substrate or substrate-ubiquitin in solution have been set up. FRET (fluorescence resonance energy transfer) assays that are routinely used in HTS have been employed for inhibitor screens. As a read out for protein ubiquitination or protein-protein interaction, the substrate of interest and ubiquitin or E3, respectively, are labelled with chromophores. One molecule is acting as the donor and the other as the acceptor; only when the two proteins and therefore the two chromophores are in close proximity can energy be transferred from the donor to the acceptor chromophore leading to emission from both the donor and the acceptor upon donor excitation [217]. The ratio between donor and acceptor emission can be used as a measure of protein-protein interaction and thus as a read-out of the efficiency of substrate modification with ubiquitin or E3-substrate interaction. In addition to FRET assays, a fluorescent polarisation based assay has been utilised where the displacement of an E3 ligase interacting fluorescein labelled peptide is determined as a measure of the E3 – substrate interaction efficiency. The technique was used to identify an allosteric inhibitor of the yeast F-box E3 ligase Cdc4; the inhibitor blocks substrate interaction and thus CDC4 mediated ubiquitination [205].

Alternatively, techniques where the ubiquitinated component of the reaction is immobilised on a microtiter plate have been developed. As most E3 ligases possess autoubiquitination activity, this is commonly used as a measure of the enzymes catalytic activity to simplify the reaction. After the ubiquitination reaction has taken place all additional components of the reaction are washed away and the amount of ubiquitin attached to the E3 ligase or substrate is quantified. The amount of ubiquitin is determined using an antibody that detects ubiquitin, either directly or by an ubiquitin-fused tag e.g. FLAG. The ubiquitin antibody is coupled to a system that allows quantification i.e. HRP (Horse Radish Peroxidase) or an ORIGEN-tag [218, 219]. In addition to immobilisation on a microtiter plate, beads can be used. The alpha screen technology for example, allows immobilisation of two proteins that carry a distinctive protein tag, e.g. His6 and GST (Glutathione S-Transferase). One protein is immobilised on the donor and the other on the acceptor alpha screen beads. Upon illumination the donor beads are able to convert oxygen to reactive singlet oxygen, which can excite acceptor beads in close proximity (up to 200 nm) to emit light at a specific wavelength and this can be detected [220]. The advantage of this technique is that, whilst immobilised, all components are present in solution. The technique has been used to screen for drugs that interfere with substrate-E3 binding [202], but can also be used to directly measure ubiquitination efficiency if ubiquitin is immobilised on the beads. As compounds identified by any of these *in vitro* methods to block ubiquitination could potentially act on any of the three enzymes involved in the reaction, i.e. E1, E2 and E3, it is important to test any hits for their inhibitory potential towards the E1 and E2 enzymes employed in the reaction to select inhibitors that specifically target the E3 enzyme.

### In cells

So far only one HTS screen for E3 ligase inhibitors utilising a cell-based assay has been reported. The study that aimed to identify inhibitors of MDM2 E3 ligase activity took advantage of the fact that MDM2 possesses autoubiquitination activity that leads to its degradation. Using MDM2-luciferease fusion proteins the levels of MDM2 protein in response to drugs from a library consisting of 270,080 compounds was evaluated by measuring luminescence. In parallel the effects of the drugs on a mutant protein MDM2-(C464) that does not exhibit E3 ligase activity was determined. Compounds that reduced levels of wild-type MDM2 but not the mutant form were selected as the authors reasoned that the reduction, in these cases, must be due to alteration of MDM2's E3 ligase activity and not its transcription, translation or regulation by other E3 ligases [221]. The advantage of this approach is that active compounds identified by the technique should be specific to the E3 ligase and not E1 or E2 enzymes. Additionally, the selected compounds are known to be active in a cellular environment; however a catalytically deficient E3 ligase mutant is required.

### HECT E3 ligases as drug targets

While the possibility to exploit the UPS and E3 ligases has been studied extensively in recent years, HECT E3 have received surprisingly little attention as potential drug targets, despite the fact that several HECT E3s have been implicated in the pathology of a range of different human diseases [10, 222, 223] (see table 2) and that they are expected to be easier to target than RING and U-Box E3s due to their catalytic centre. HECT enzymes were the first E3s to be discovered in 1995 [22]; subsequently a total of twenty-eight human and five yeast HECT E3s have been identified [222]. HECT E3 ligases are characterised by a C-terminal HECT domain consisting of ~350 amino acids, which, unlike RING and RING type domains, contains intrinsic catalytic activity. The HECT domain interacts with ubiquitin charged E2 enzymes, primarily members of the UbcH5 family and UbcH7. Upon interaction, the ubiquitin molecule associated with the E2 forms a thioesther bond with a conserved cysteine in the catalytic centre of the HECT domain. Next, the ubiquitin is transferred onto a lysine residue in the target protein; this can be either a substrate of the E3, an ubiquitin molecule (chain elongation) or the E3 itself (autoubiquitination). While the C-terminus is required for its catalytic activity and E2 interactions, the N-terminal part of these proteins is mainly responsible for substrate interactions and thus determines the specificity of the enzyme [222]. While the linkage and length of ubiquitin chains catalysed by RING type E3s depends on the specific E2/E3 pair, in the case of HECT E3s chain specificity is mainly provided by the E3 ligase alone – making the outcome of HECT E3-mediated ubiquitination more predictable than that of RING E3s. However, how chain specificity is achieved mechanistically remains to be investigated in detail, although it appears to be a combination of substrate and binding partners [222]. There are three main families of HECT E3 based on the N-terminal region of the enzymes: Nedd4 (9 members), HERC (6 members) and other HECTs (13 members) [224-226]. Deregulation of HECT E3s was shown to play an important role in the development of several different kinds of human pathologies, including cancer [10, 59, 227] and neurodegeneration [228, 229] (see table 2 for list of HECT E3s implicated in human diseases). As we are developing a better understanding of the role of different HECT proteins and their involvement in human diseases, small molecular modulators of HECT E3 activity could provide powerful tools in the treatment of HECT E3-associated diseases.

**Table 2:** HECT E3 ligases in human pathologies

E3 ligase	Implicated in disease	Reference
**Nedd4**	Developmental defects, Vascular defects, denervation-induced skeletal muscle atrophy, Neuroblastoma and pancreatic cancer, budding of viruses	[241-251]
**NEDD4-2**	Liddle's syndrome, hypertension	[252-254]
**Itch**	Inflammatory diseases, Cancer development	[59, 89, 227, 255]
**WWP1**	Breast and prostate cancer, Cancer cell migration and metastasis, (MSC differentiation)	[254]
**WWP2**	Diseases of iron homeostasis (hemochromatosis and anemia). Regulates tumour suppressor PTEN	[63, 256-258] [259]
**SMURF1/SMURF2**	Cancer, involved in cell proliferation, DNA damage response and tumour suppression	[224, 260, 261]
**NEDL1/HECW1 and NEDL2/HECW2**	Familial amyotrophic sclerosis (FALS), Neurodegenerative diseases, NEDL2 binds to and stabilises p73	[63, 256-258, 262-266]
**HERC1**	Tuberous sclerosis complex (TSC), Acute lymphoblastic leukemia, Sporadic colorectal cancer	[260, 261]
**HERC2**	DNA damage response and repair, neurodevelopmental disorders	[263-265, 267-270]
**HERC5**	Antiviral response	[222, 266, 271-275]
**E6AP**	Angelman syndrome, Cervical cancer, Autism spectrum disorder	[267-270, 276-278]
**HUWE1**	Cell Proliferation and apoptosis, DNA repair, neuronal differentiation, cancer development	[222, 273-275, 279]
**EDD/UBR5**	Breast and ovarian cancer	[277-281]
**TRIP12/ULF**	Acute myeloid leukemia	[279]
**HACE1**	Wilms' tumours, neuroblastom, Huntington's disease	[223, 279-281]

**Figure 4:**
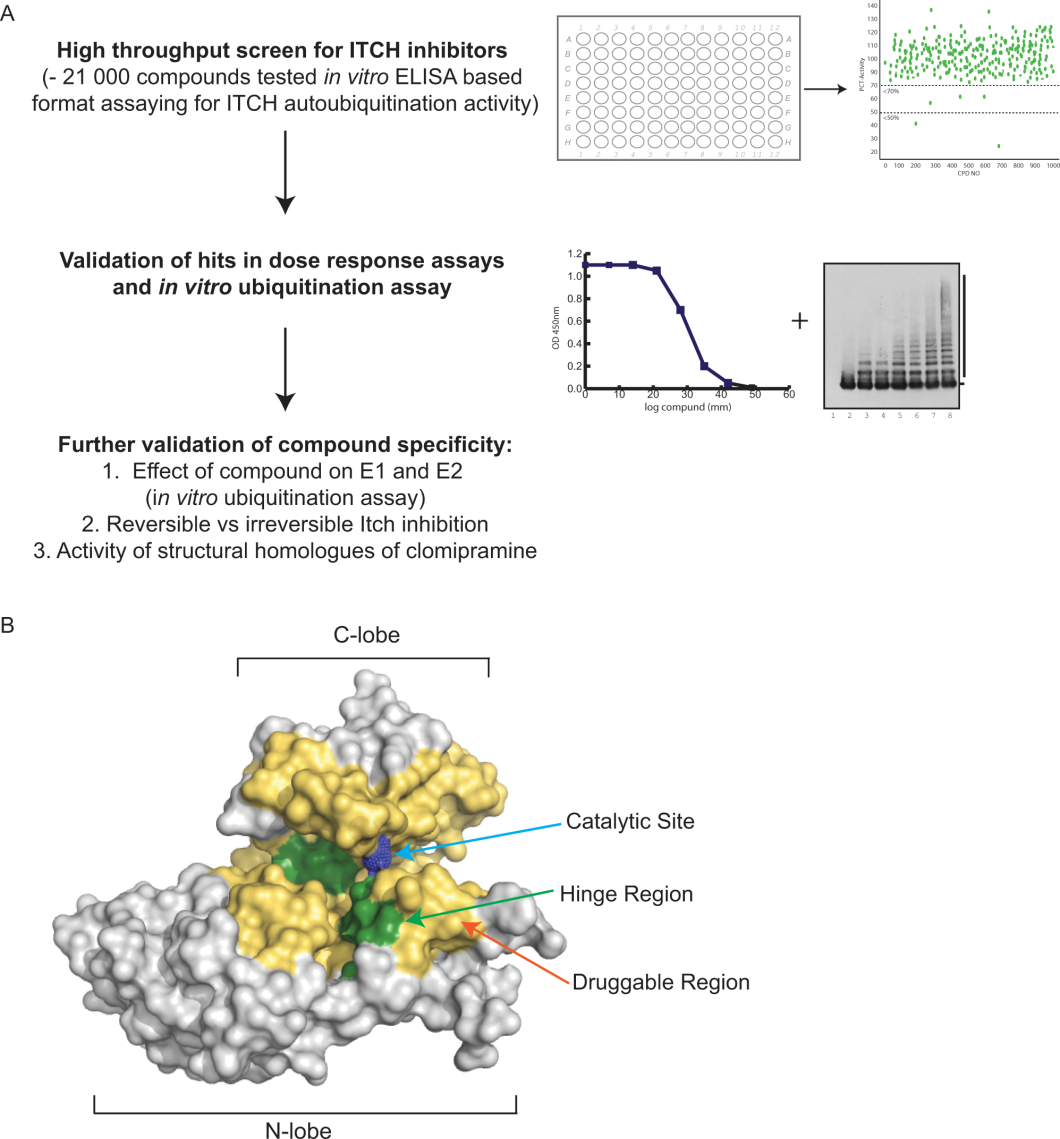
Development of an Itch inhibitor (A) Flowchart of a high throughput screen to identify Itch inhibitors. The screen was carried out using an ELISA based assay with Itch immobilised on the plate and all other components of the ubiquitination reaction in solution. Itch ubiquitination was determined using an antibody detecting poly-ubiquitin. The effect of ~21 000 compounds on Itch autoubiquitination was determined. Compounds that showed an inhibitory effect were further validated using dose response curves of the ELISA based assay and *in vitro* ubiquitination assays with the Itch substrate p73. In a further validation step an effect of the identified compound, clomipramine, on the E1 and E2 enzymes was ruled out and structural homologues of clomipramine were analysed for Itch inhibitory activity. ELISA= enzyme-linked immunosorbent assay. (B) Drugable surface of Itch and predicted Clomipramine binding sites. The active cysteine of the HECT ligase Itch is shown as blue spheres and the predicated drugable area, based on electrostatics and proximity to the active cysteine, is shown in yellow. The binding site of clomipramine was predicted using docking programs and the two binding sites that exhibit low-energy conformations are shown in green.

A paper recently published by our group [230] demonstrated the first high throughput screen for small molecular weight inhibitors of the HECT E3 ligase Itch. Using an ELISA based HTS screen, the ability of ~20,000 compounds to inhibit Itch autoubiquitination was determined (Figure 4A). The screen showed that a comparably low proportion of these small molecules possessed inhibitor activity, and identified only six hits that exhibited dose-dependent inhibition of Itch. The compounds were further tested towards their potential to inhibit Itch-mediated substrate ubiquitination, and only one compound was shown to inhibit Itch autoubiquitination and p73 ubiquitination. Further analysis of the inhibitor showed that while it had no inhibitory effect on two RING E3s, Diap and RING1B, it prevented autoubiquitination of the HECT E3 E6AP and thus appears specific for this sub-family of E3s. The small molecule compound identified through this approach was clomipramine, a clinically useful antidepressant drug. The effects in cancer cells treated with clomipramine included reduction of cell growth, and synergism with gemcitabine or mitomycin in killing cancer cells through autophagy blockade [230]. Because clomipramine binds Itch in an irreversible manner and can affect other HECT E3s, it is possible that it forms a covalent bond with the catalytic cysteine found in the HECT domain [230]. Interestingly, we have identified two other possible binding sites in the HECT domain that may accommodate clomipramine (Figure 4B) [230].

We have previously shown that clomipramine interferes with autophagic flux [63] in what appears to be unrelated to its Itch inhibitory activity. This demonstrates the issue of polypharmacology, which adds to the complexity of targeting specific proteins for degradation. Even if a drug exhibits specificity towards a certain E3, it might display a number of “off-target effects” that are very difficult to predict. Furthermore, Itch is known to target other proteins for ubiquitination [231] and is linked to important cellular pathways such as Hippo [72, 226] adding to the complexity of predicting the consequence of Itch inhibition *in vivo*.

Another approach to identify HECT E3 ligase inhibitors was taken by Yamagishi *et al*. [232], which identified an N-methyl-peptide that inhibits activity of E6AP. The group developed a technique to synthesize a de novo library of ‘natural product like’ nonstandard peptides and a screening technique called RaPID (random nonstandard peptides integrated discovery), where every peptide is linked to its respective mRNA sequence via puromycin. Using an *in vitro* display technique, peptides that bind to the E6AP HECT domain were identified and one was further validated as an inhibitor of E6AP E3 ligase activity in *in vitro* ubiquitination assays [232]. These studies are thus a proof of concept for identification of HECT E3 ligase specific inhibitor by *in vitro* HTS. Further validation and development of these and/or other compounds will be necessary to show if HECT E3 ligase inhibition can be exploited therapeutically in the clinic.

## CONCLUDING REMARKS

E3 ligases are key molecules in the regulation of protein degradation, activation and localisation and as such are promising targets for therapeutic intervention [233]. Importantly, E3 ligases are tight regulators of transcription factors; another class of key molecules that are known to be deregulated in many disease types and that are considered undrugable. Indeed, efforts are underway to develop small molecules that target E3 ligases in order to manipulate the activity of transcription factors. How does one find a small molecule that will inhibit an E3 ligase? Naturally, there are several strategies that can be adopted although inevitably this is challenging due to the complex nature of the ubiquitination reaction. Moreover, it is hard to predict how a small molecule identified to block an E3 *in vitro* will function *in vivo* due to the fact that each E3 ligase is likely to ubiquitinate several different proteins.

The success of the personalized medicine approach depends on the availability of compounds that can hit the identified pathway found to be important in a given patient context. Therefore, a collection of small molecules that can manipulate key cellular elements including E3 ligases and transcription factors is essential. Indeed, the growing number of specific E3 ligase inhibitors (table 1) under development is encouraging and may lead to the first E3 ligase inhibitor approved for clinical use.

## ABBREVIATIONS

TA: transcriptional activator
ATP: adenosine triphosphate
AMP: adenosine monophosphate
SUMO: small ubiquitin-related modifier
NLS: nuclear localisation sequence
FDA: U.S. Food and Drug Administration
Ub: ubiquitin

## References

[1] Melino G, Knight RA, Nicotera P (2005). How many ways to die? How many different models of cell death?. Cell death and differentiation.

[2] Muller M, Schleithoff ES, Stremmel W, Melino G, Krammer PH, Schilling T (2006). One, two, three--p53, p63, p73 and chemosensitivity. Drug resistance updates : reviews and commentaries in antimicrobial and anticancer chemotherapy.

[3] Ciechanover A (2005). Intracellular protein degradation: from a vague idea thru the lysosome and the ubiquitin-proteasome system and onto human diseases and drug targeting. Cell death and differentiation.

[4] Hershko A (2005). The ubiquitin system for protein degradation and some of its roles in the control of the cell division cycle. Cell death and differentiation.

[5] Rose I (2005). Ubiquitin at Fox Chase. Cell death and differentiation.

[6] Severe N, Dieudonne FX, Marie PJ (2013). E3 ubiquitin ligase-mediated regulation of bone formation and tumorigenesis. Cell death & disease.

[7] Smalle J, Vierstra RD (2004). The ubiquitin 26S proteasome proteolytic pathway. Annual review of plant biology.

[8] Bernassola F, Ciechanover A, Melino G (2010). The ubiquitin proteasome system and its involvement in cell death pathways. Cell death and differentiation.

[9] Shi D, Grossman SR (2010). Ubiquitin becomes ubiquitous in cancer: emerging roles of ubiquitin ligases and deubiquitinases in tumorigenesis and as therapeutic targets. Cancer biology & therapy.

[10] Bernassola F, Karin M, Ciechanover A, Melino G (2008). The HECT family of E3 ubiquitin ligases: multiple players in cancer development. Cancer cell.

[11] Schwartz AL, Ciechanover A (2009). Targeting proteins for destruction by the ubiquitin system: implications for human pathobiology. Annual review of pharmacology and toxicology.

[12] Halaby MJ, Hakem R, Hakem A (2013). Pirh2: an E3 ligase with central roles in the regulation of cell cycle, DNA damage response, and differentiation. Cell cycle (Georgetown, Tex).

[13] Navon A, Ciechanover A (2009). The 26 S proteasome: from basic mechanisms to drug targeting. The Journal of biological chemistry.

[14] Cohen P, Tcherpakov M (2010). Will the ubiquitin system furnish as many drug targets as protein kinases?. Cell.

[15] Davare MA, Saborowski A, Eide CA, Tognon C, Smith RL, Elferich J, Agarwal A, Tyner JW, Shinde UP, Lowe SW, Druker BJ (2013). Foretinib is a potent inhibitor of oncogenic ROS1 fusion proteins. Proceedings of the National Academy of Sciences of the United States of America.

[16] Orlowski RZ, Stinchcombe TE, Mitchell BS, Shea TC, Baldwin AS, Stahl S, Adams J, Esseltine DL, Elliott PJ, Pien CS, Guerciolini R, Anderson JK, Depcik-Smith ND, Bhagat R, Lehman MJ, Novick SC (2002). Phase I trial of the proteasome inhibitor PS-341 in patients with refractory hematologic malignancies. Journal of clinical oncology : official journal of the American Society of Clinical Oncology.

[17] Haas AL, Rose IA (1982). The mechanism of ubiquitin activating enzyme. A kinetic and equilibrium analysis. The Journal of biological chemistry.

[18] Haas AL, Warms JV, Hershko A, Rose IA (1982). Ubiquitin-activating enzyme. Mechanism and role in protein-ubiquitin conjugation. The Journal of biological chemistry.

[19] Hershko A, Ciechanover A (1998). The ubiquitin system. Annual review of biochemistry.

[20] Pickart CM, Eddins MJ (2004). Ubiquitin: structures, functions, mechanisms. Biochimica et biophysica acta.

[21] Nuber U, Schwarz S, Kaiser P, Schneider R, Scheffner M (1996). Cloning of human ubiquitin-conjugating enzymes UbcH6 and UbcH7 (E2-F1) and characterization of their interaction with E6-AP and RSP5. The Journal of biological chemistry.

[22] Huibregtse JM, Scheffner M, Beaudenon S, Howley PM (1995). A family of proteins structurally and functionally related to the E6-AP ubiquitin-protein ligase. Proceedings of the National Academy of Sciences of the United States of America.

[23] Li Y, Kong Y, Zhou Z, Chen H, Wang Z, Hsieh YC, Zhao D, Zhi X, Huang J, Zhang J, Li H, Chen C (2013). The HECTD3 E3 ubiquitin ligase facilitates cancer cell survival by promoting K63-linked polyubiquitination of caspase-8. Cell death & disease.

[24] Zhao Y, Xiong X, Jia L, Sun Y (2012). Targeting Cullin-RING ligases by MLN4924 induces autophagy via modulating the HIF1-REDD1-TSC1-mTORC1-DEPTOR axis. Cell death & disease.

[25] Li Y, Kong Y, Zhou Z, Chen H, Wang Z, Hsieh YC, Zhao D, Zhi X, Huang J, Zhang J, Li H, Chen C (2013). The HECTD3 E3 ubiquitin ligase facilitates cancer cell survival by promoting K63-linked polyubiquitination of caspase-8. Cell death & disease.

[26] Mevissen TE, Hospenthal MK, Geurink PP, Elliott PR, Akutsu M, Arnaudo N, Ekkebus R, Kulathu Y, Wauer T, El Oualid F, Freund SM, Ovaa H, Komander D (2013). OTU deubiquitinases reveal mechanisms of linkage specificity and enable ubiquitin chain restriction analysis. Cell.

[27] Faronato M, Patel V, Darling S, Dearden L, Clague MJ, Urbe S, Coulson JM (2013). The deubiquitylase USP15 stabilizes newly synthesized REST and rescues its expression at mitotic exit. Cell cycle (Georgetown, Tex).

[28] Yang CS, Sinenko SA, Thomenius MJ, Robeson AC, Freel CD, Horn SR, Kornbluth S (2014). The deubiquitinating enzyme DUBAI stabilizes DIAP1 to suppress Drosophila apoptosis. Cell death and differentiation.

[29] Thrower JS, Hoffman L, Rechsteiner M, Pickart CM (2000). Recognition of the polyubiquitin proteolytic signal. The EMBO journal.

[30] Suryadinata R, Holien JK, Yang G, Parker MW, Papaleo E, Sarcevic B (2013). Molecular and structural insight into lysine selection on substrate and ubiquitin lysine 48 by the ubiquitin-conjugating enzyme Cdc34. Cell cycle (Georgetown, Tex).

[31] Kirisako T, Kamei K, Murata S, Kato M, Fukumoto H, Kanie M, Sano S, Tokunaga F, Tanaka K, Iwai K (2006). A ubiquitin ligase complex assembles linear polyubiquitin chains. The EMBO journal.

[32] Gerlach B, Cordier SM, Schmukle AC, Emmerich CH, Rieser E, Haas TL, Webb AI, Rickard JA, Anderton H, Wong WW, Nachbur U, Gangoda L, Warnken U, Purcell AW, Silke J, Walczak H (2011). Linear ubiquitination prevents inflammation and regulates immune signalling. Nature.

[33] Ben-Saadon R, Zaaroor D, Ziv T, Ciechanover A (2006). The polycomb protein Ring1B generates self atypical mixed ubiquitin chains required for its in vitro histone H2A ligase activity. Molecular cell.

[34] Komander D, Rape M (2012). The ubiquitin code. Annual review of biochemistry.

[35] Xu P, Duong DM, Seyfried NT, Cheng D, Xie Y, Robert J, Rush J, Hochstrasser M, Finley D, Peng J (2009). Quantitative proteomics reveals the function of unconventional ubiquitin chains in proteasomal degradation. Cell.

[36] Bedford L, Layfield R, Mayer RJ, Peng J, Xu P (2011). Diverse polyubiquitin chains accumulate following 26S proteasomal dysfunction in mammalian neurones. Neuroscience letters.

[37] Kravtsova-Ivantsiv Y, Ciechanover A (2012). Non-canonical ubiquitin-based signals for proteasomal degradation. Journal of cell science.

[38] Jacobson AD, Zhang NY, Xu P, Han KJ, Noone S, Peng J, Liu CW (2009). The lysine 48 and lysine 63 ubiquitin conjugates are processed differently by the 26 s proteasome. The Journal of biological chemistry.

[39] Strieter ER, Korasick DA (2012). Unraveling the complexity of ubiquitin signaling. ACS chemical biology.

[40] Lauwers E, Erpapazoglou Z, Haguenauer-Tsapis R, Andre B (2010). The ubiquitin code of yeast permease trafficking. Trends in cell biology.

[41] Lauwers E, Jacob C, Andre B (2009). K63-linked ubiquitin chains as a specific signal for protein sorting into the multivesicular body pathway. The Journal of cell biology.

[42] Shih SC, Sloper-Mould KE, Hicke L (2000). Monoubiquitin carries a novel internalization signal that is appended to activated receptors. The EMBO journal.

[43] Terrell J, Shih S, Dunn R, Hicke L (1998). A function for monoubiquitination in the internalization of a G protein-coupled receptor. Molecular cell.

[44] Nakatsu F, Sakuma M, Matsuo Y, Arase H, Yamasaki S, Nakamura N, Saito T, Ohno H (2000). A Di-leucine signal in the ubiquitin moiety. Possible involvement in ubiquitination-mediated endocytosis. The Journal of biological chemistry.

[45] Haglund K, Sigismund S, Polo S, Szymkiewicz I, Di Fiore PP, Dikic I (2003). Multiple monoubiquitination of RTKs is sufficient for their endocytosis and degradation. Nature cell biology.

[46] Mosesson Y, Shtiegman K, Katz M, Zwang Y, Vereb G, Szollosi J, Yarden Y (2003). Endocytosis of receptor tyrosine kinases is driven by monoubiquitylation, not polyubiquitylation. The Journal of biological chemistry.

[47] Kirkin V, McEwan DG, Novak I, Dikic I (2009). A role for ubiquitin in selective autophagy. Molecular cell.

[48] Shaid S, Brandts CH, Serve H, Dikic I (2013). Ubiquitination and selective autophagy. Cell death and differentiation.

[49] Pal P, Lochab S, Kanaujiya JK, Kapoor I, Sanyal S, Behre G, Trivedi AK (2013). E6AP, an E3 ubiquitin ligase negatively regulates granulopoiesis by targeting transcription factor C/EBP alpha for ubiquitin-mediated proteasome degradation. Cell death & disease.

[50] Alkalay I, Yaron A, Hatzubai A, Orian A, Ciechanover A, Ben-Neriah Y (1995). Stimulation-dependent I kappa B alpha phosphorylation marks the NF-kappa B inhibitor for degradation via the ubiquitin-proteasome pathway. Proceedings of the National Academy of Sciences of the United States of America.

[51] Hayden MS, Ghosh S (2008). Shared principles in NF-kappaB signaling. Cell.

[52] Kaelin WG (2008). The von Hippel-Lindau tumour suppressor protein: O2 sensing and cancer. Nature reviews Cancer.

[53] Tian YM, Yeoh KK, Lee MK, Eriksson T, Kessler BM, Kramer HB, Edelmann MJ, Willam C, Pugh CW, Schofield CJ, Ratcliffe PJ (2011). Differential sensitivity of hypoxia inducible factor hydroxylation sites to hypoxia and hydroxylase inhibitors. The Journal of biological chemistry.

[54] Rotili D, Altun M, Kawamura A, Wolf A, Fischer R, Leung IK, Mackeen MM, Tian YM, Ratcliffe PJ, Mai A, Kessler BM, Schofield CJ (2011). A photoreactive small-molecule probe for 2-oxoglutarate oxygenases. Chemistry & biology.

[55] Leveillard T, Gorry P, Niederreither K, Wasylyk B (1998). MDM2 expression during mouse embryogenesis and the requirement of p53. Mechanisms of development.

[56] Montes de Oca Luna R, Wagner DS, Lozano G (1995). Rescue of early embryonic lethality in mdm2-deficient mice by deletion of p53. Nature.

[57] Parant J, Chavez-Reyes A, Little NA, Yan W, Reinke V, Jochemsen AG, Lozano G (2001). Rescue of embryonic lethality in Mdm4-null mice by loss of Trp53 suggests a nonoverlapping pathway with MDM2 to regulate p53. Nature genetics.

[58] Love IM, Grossman SR (2012). It Takes 15 to Tango: Making Sense of the Many Ubiquitin Ligases of p53. Genes & cancer.

[59] Melino G, Gallagher E, Aqeilan RI, Knight R, Peschiaroli A, Rossi M, Scialpi F, Malatesta M, Zocchi L, Browne G, Ciechanover A, Bernassola F (2008). Itch: a HECT-type E3 ligase regulating immunity, skin and cancer. Cell death and differentiation.

[60] Yang A, Schweitzer R, Sun D, Kaghad M, Walker N, Bronson RT, Tabin C, Sharpe A, Caput D, Crum C, McKeon F (1999). p63 is essential for regenerative proliferation in limb, craniofacial and epithelial development. Nature.

[61] Mills AA, Zheng B, Wang XJ, Vogel H, Roop DR, Bradley A (1999). p63 is a p53 homologue required for limb and epidermal morphogenesis. Nature.

[62] Agostini M, Tucci P, Killick R, Candi E, Sayan BS, Rivetti di Val Cervo P, Nicotera P, McKeon F, Knight RA, Mak TW, Melino G (2011). Neuronal differentiation by TAp73 is mediated by microRNA-34a regulation of synaptic protein targets. Proceedings of the National Academy of Sciences of the United States of America.

[63] Rossi M, Munarriz ER, Bartesaghi S, Milanese M, Dinsdale D, Guerra-Martin MA, Bampton ET, Glynn P, Bonanno G, Knight RA, Nicotera P, Melino G (2009). Desmethylclomipramine induces the accumulation of autophagy markers by blocking autophagic flux. Journal of cell science.

[64] Bellomaria A, Barbato G, Melino G, Paci M, Melino S (2010). Recognition of p63 by the E3 ligase ITCH: Effect of an ectodermal dysplasia mutant. Cell cycle (Georgetown, Tex).

[65] Bellomaria A, Barbato G, Melino G, Paci M, Melino S (2012). Recognition mechanism of p63 by the E3 ligase Itch: novel strategy in the study and inhibition of this interaction. Cell cycle (Georgetown, Tex).

[66] Melino G, Knight RA, Cesareni G (2006). Degradation of p63 by Itch. Cell cycle (Georgetown, Tex).

[67] Rossi M, Aqeilan RI, Neale M, Candi E, Salomoni P, Knight RA, Croce CM, Melino G (2006). The E3 ubiquitin ligase Itch controls the protein stability of p63. Proceedings of the National Academy of Sciences of the United States of America.

[68] Rossi M, De Laurenzi V, Munarriz E, Green DR, Liu YC, Vousden KH, Cesareni G, Melino G (2005). The ubiquitin-protein ligase Itch regulates p73 stability. The EMBO journal.

[69] Eldridge AG, O'Brien T (2010). Therapeutic strategies within the ubiquitin proteasome system. Cell death and differentiation.

[70] Levy D, Adamovich Y, Reuven N, Shaul Y (2007). The Yes-associated protein 1 stabilizes p73 by preventing Itch-mediated ubiquitination of p73. Cell death and differentiation.

[71] Levy D, Reuven N, Shaul Y (2008). A regulatory circuit controlling Itch-mediated p73 degradation by Runx. The Journal of biological chemistry.

[72] Oberst A, Malatesta M, Aqeilan RI, Rossi M, Salomoni P, Murillas R, Sharma P, Kuehn MR, Oren M, Croce CM, Bernassola F, Melino G (2007). The Nedd4-binding partner 1 (N4BP1) protein is an inhibitor of the E3 ligase Itch. Proceedings of the National Academy of Sciences of the United States of America.

[73] Aqeilan RI, Pekarsky Y, Herrero JJ, Palamarchuk A, Letofsky J, Druck T, Trapasso F, Han SY, Melino G, Huebner K, Croce CM (2004). Functional association between Wwox tumor suppressor protein and p73, a p53 homolog. Proceedings of the National Academy of Sciences of the United States of America.

[74] Salah Z, Bar-mag T, Kohn Y, Pichiorri F, Palumbo T, Melino G, Aqeilan RI (2013). Tumor suppressor WWOX binds to DeltaNp63alpha and sensitizes cancer cells to chemotherapy. Cell death & disease.

[75] Peschiaroli A, Scialpi F, Bernassola F, El Sherbini E, Melino G (2010). The E3 ubiquitin ligase WWP1 regulates Delta Np63-dependent transcription through Lys63 linkages. Biochem Bioph Res Co.

[76] Li C, Chang DL, Yang Z, Qi J, Liu R, He H, Li D, Xiao ZX (2013). Pin1 modulates p63alpha protein stability in regulation of cell survival, proliferation and tumor formation. Cell death & disease.

[77] Sayan BS, Yang AL, Conforti F, Tucci P, Piro MC, Browne GJ, Agostini M, Bernardini S, Knight RA, Mak TW, Melino G (2010). Differential control of TAp73 and DeltaNp73 protein stability by the ring finger ubiquitin ligase PIR2. Proceedings of the National Academy of Sciences of the United States of America.

[78] Peschiaroli A, Scialpi F, Bernassola F, Pagano M, Melino G (2009). The F-box protein FBXO45 promotes the proteasome-dependent degradation of p73. Oncogene.

[79] Gonzalez-Cano L, Hillje AL, Fuertes-Alvarez S, Marques MM, Blanch A, Ian RW, Irwin MS, Schwamborn JC, Marin MC (2013). Regulatory feedback loop between TP73 and TRIM32. Cell death & disease.

[80] Chen ZJ, Sun LJ (2009). Nonproteolytic functions of ubiquitin in cell signaling. Molecular cell.

[81] Dikic I, Wakatsuki S, Walters KJ (2009). Ubiquitin-binding domains - from structures to functions. Nature reviews Molecular cell biology.

[82] Hochstrasser M (2009). Origin and function of ubiquitin-like proteins. Nature.

[83] Baker R, Lewis SM, Sasaki AT, Wilkerson EM, Locasale JW, Cantley LC, Kuhlman B, Dohlman HG, Campbell SL (2013). Site-specific monoubiquitination activates Ras by impeding GTPase-activating protein function. Nature structural & molecular biology.

[84] Chernorudskiy AL, Gainullin MR (2013). Ubiquitin system: direct effects join the signaling. Science signaling.

[85] Sagar GD, Gereben B, Callebaut I, Mornon JP, Zeold A, da Silva WS, Luongo C, Dentice M, Tente SM, Freitas BC, Harney JW, Zavacki AM, Bianco AC (2007). Ubiquitination-induced conformational change within the deiodinase dimer is a switch regulating enzyme activity. Molecular and cellular biology.

[86] Chen BB, Mallampalli RK (2009). Masking of a nuclear signal motif by monoubiquitination leads to mislocalization and degradation of the regulatory enzyme cytidylyltransferase. Molecular and cellular biology.

[87] Lee JT, Gu W (2010). The multiple levels of regulation by p53 ubiquitination. Cell death and differentiation.

[88] Green DR, Kroemer G (2009). Cytoplasmic functions of the tumour suppressor p53. Nature.

[89] Hansen TM, Rossi M, Roperch JP, Ansell K, Simpson K, Taylor D, Mathon N, Knight RA, Melino G (2007). Itch inhibition regulates chemosensitivity in vitro. Biochem Biophys Res Commun.

[90] Fukuchi M, Imamura T, Chiba T, Ebisawa T, Kawabata M, Tanaka K, Miyazono K (2001). Ligand-dependent degradation of Smad3 by a ubiquitin ligase complex of ROC1 and associated proteins. Molecular biology of the cell.

[91] Trotman LC, Wang X, Alimonti A, Chen Z, Teruya-Feldstein J, Yang H, Pavletich NP, Carver BS, Cordon-Cardo C, Erdjument-Bromage H, Tempst P, Chi SG, Kim HJ, Misteli T, Jiang X, Pandolfi PP (2007). Ubiquitination regulates PTEN nuclear import and tumor suppression. Cell.

[92] Geng F, Wenzel S, Tansey WP (2012). Ubiquitin and proteasomes in transcription. Annual review of biochemistry.

[93] Salghetti SE, Kim SY, Tansey WP (1999). Destruction of Myc by ubiquitin-mediated proteolysis: cancer-associated and transforming mutations stabilize Myc. The EMBO journal.

[94] Salghetti SE, Muratani M, Wijnen H, Futcher B, Tansey WP (2000). Functional overlap of sequences that activate transcription and signal ubiquitin-mediated proteolysis. Proceedings of the National Academy of Sciences of the United States of America.

[95] Archer CT, Burdine L, Liu B, Ferdous A, Johnston SA, Kodadek T (2008). Physical and functional interactions of monoubiquitylated transactivators with the proteasome. The Journal of biological chemistry.

[96] Bres V, Kiernan RE, Linares LK, Chable-Bessia C, Plechakova O, Treand C, Emiliani S, Peloponese JM, Jeang KT, Coux O, Scheffner M, Benkirane M (2003). A non-proteolytic role for ubiquitin in Tat-mediated transactivation of the HIV-1 promoter. Nature cell biology.

[97] Gammoh N, Gardiol D, Massimi P, Banks L (2009). The Mdm2 ubiquitin ligase enhances transcriptional activity of human papillomavirus E2. Journal of virology.

[98] Greer SF, Zika E, Conti B, Zhu XS, Ting JP (2003). Enhancement of CIITA transcriptional function by ubiquitin. Nature immunology.

[99] Brenkman AB, de Keizer PL, van den Broek NJ, Jochemsen AG, Burgering BM (2008). Mdm2 induces mono-ubiquitination of FOXO4. PloS one.

[100] Thomas D, Tyers M (2000). Transcriptional regulation: Kamikaze activators. Current biology : CB.

[101] Salghetti SE, Caudy AA, Chenoweth JG, Tansey WP (2001). Regulation of transcriptional activation domain function by ubiquitin. Science (New York, NY).

[102] Molineaux SM (2012). Molecular pathways: targeting proteasomal protein degradation in cancer. Clinical cancer research : an official journal of the American Association for Cancer Research.

[103] Kawabata S, Gills JJ, Mercado-Matos JR, Lopiccolo J, Wilson W, Hollander MC, Dennis PA (2012). Synergistic effects of nelfinavir and bortezomib on proteotoxic death of NSCLC and multiple myeloma cells. Cell death & disease.

[104] Lichtenberg M, Mansilla A, Zecchini VR, Fleming A, Rubinsztein DC (2011). The Parkinson's disease protein LRRK2 impairs proteasome substrate clearance without affecting proteasome catalytic activity. Cell death & disease.

[105] Hideshima T, Richardson P, Chauhan D, Palombella VJ, Elliott PJ, Adams J, Anderson KC (2001). The proteasome inhibitor PS-341 inhibits growth, induces apoptosis, and overcomes drug resistance in human multiple myeloma cells. Cancer research.

[106] Adams J (2004). The development of proteasome inhibitors as anticancer drugs. Cancer cell.

[107] Fribley A, Zeng Q, Wang CY (2004). Proteasome inhibitor PS-341 induces apoptosis through induction of endoplasmic reticulum stress-reactive oxygen species in head and neck squamous cell carcinoma cells. Molecular and cellular biology.

[108] Hideshima T, Chauhan D, Richardson P, Mitsiades C, Mitsiades N, Hayashi T, Munshi N, Dang L, Castro A, Palombella V, Adams J, Anderson KC (2002). NF-kappa B as a therapeutic target in multiple myeloma. The Journal of biological chemistry.

[109] Williams S, Pettaway C, Song R, Papandreou C, Logothetis C, McConkey DJ (2003). Differential effects of the proteasome inhibitor bortezomib on apoptosis and angiogenesis in human prostate tumor xenografts. Molecular cancer therapeutics.

[110] Neri P, Ren L, Gratton K, Stebner E, Johnson J, Klimowicz A, Duggan P, Tassone P, Mansoor A, Stewart DA, Lonial S, Boise LH, Bahlis NJ (2011). Bortezomib-induced “BRCAness” sensitizes multiple myeloma cells to PARP inhibitors. Blood.

[111] Nowis D, Maczewski M, Mackiewicz U, Kujawa M, Ratajska A, Wieckowski MR, Wilczynski GM, Malinowska M, Bil J, Salwa P, Bugajski M, Wojcik C, Sinski M, Abramczyk P, Winiarska M, Dabrowska-Iwanicka A (2010). Cardiotoxicity of the anticancer therapeutic agent bortezomib. The American journal of pathology.

[112] Oerlemans R, Franke NE, Assaraf YG, Cloos J, van Zantwijk I, Berkers CR, Scheffer GL, Debipersad K, Vojtekova K, Lemos C, van der Heijden JW, Ylstra B, Peters GJ, Kaspers GL, Dijkmans BA, Scheper RJ (2008). Molecular basis of bortezomib resistance: proteasome subunit beta5 (PSMB5) gene mutation and overexpression of PSMB5 protein. Blood.

[113] Suzuki E, Demo S, Deu E, Keats J, Arastu-Kapur S, Bergsagel PL, Bennett MK, Kirk CJ (2011). Molecular mechanisms of bortezomib resistant adenocarcinoma cells. PloS one.

[114] Kuhn DJ, Chen Q, Voorhees PM, Strader JS, Shenk KD, Sun CM, Demo SD, Bennett MK, van Leeuwen FW, Chanan-Khan AA, Orlowski RZ (2007). Potent activity of carfilzomib, a novel, irreversible inhibitor of the ubiquitin-proteasome pathway, against preclinical models of multiple myeloma. Blood.

[115] Bhutani S, Das A, Maheshwari M, Lakhotia SC, Jana NR (2012). Dysregulation of core components of SCF complex in poly-glutamine disorders. Cell death & disease.

[116] Chatterjee A, Chatterjee U, Ghosh MK (2013). Activation of protein kinase CK2 attenuates FOXO3a functioning in a PML-dependent manner: implications in human prostate cancer. Cell death & disease.

[117] Costa AC, Loh SH, Martins LM (2013). Drosophila Trap1 protects against mitochondrial dysfunction in a PINK1/parkin model of Parkinson's disease. Cell death & disease.

[118] Gillissen B, Richter A, Richter A, Overkamp T, Essmann F, Hemmati PG, Preissner R, Belka C, Daniel PT (2013). Targeted therapy of the XIAP/proteasome pathway overcomes TRAIL-resistance in carcinoma by switching apoptosis signaling to a Bax/Bak-independent ‘type I’ mode. Cell death & disease.

[119] Kim AD, Kang KA, Kim HS, Kim DH, Choi YH, Lee SJ, Kim HS, Hyun JW (2013). A ginseng metabolite, compound K, induces autophagy and apoptosis via generation of reactive oxygen species and activation of JNK in human colon cancer cells. Cell death & disease.

[120] Pal P, Lochab S, Kanaujiya JK, Kapoor I, Sanyal S, Behre G, Trivedi AK (2013). E6AP, an E3 ubiquitin ligase negatively regulates granulopoiesis by targeting transcription factor C/EBPalpha for ubiquitin-mediated proteasome degradation. Cell death & disease.

[121] Santin I, Moore F, Grieco FA, Marchetti P, Brancolini C, Eizirik DL (2012). USP18 is a key regulator of the interferon-driven gene network modulating pancreatic beta cell inflammation and apoptosis. Cell death & disease.

[122] Wong VK, Li T, Law BY, Ma ED, Yip NC, Michelangeli F, Law CK, Zhang MM, Lam KY, Chan PL, Liu L (2013). Saikosaponin-d, a novel SERCA inhibitor, induces autophagic cell death in apoptosis-defective cells. Cell death & disease.

[123] da Silva SR, Paiva SL, Lukkarila JL, Gunning PT (2013). Exploring a new frontier in cancer treatment: targeting the ubiquitin and ubiquitin-like activating enzymes. Journal of medicinal chemistry.

[124] Yang Y, Kitagaki J, Dai RM, Tsai YC, Lorick KL, Ludwig RL, Pierre SA, Jensen JP, Davydov IV, Oberoi P, Li CC, Kenten JH, Beutler JA, Vousden KH, Weissman AM (2007). Inhibitors of ubiquitin-activating enzyme (E1), a new class of potential cancer therapeutics. Cancer research.

[125] Boggio R, Colombo R, Hay RT, Draetta GF, Chiocca S (2004). A mechanism for inhibiting the SUMO pathway. Molecular cell.

[126] Fukuda I, Ito A, Hirai G, Nishimura S, Kawasaki H, Saitoh H, Kimura K, Sodeoka M, Yoshida M (2009). Ginkgolic acid inhibits protein SUMOylation by blocking formation of the E1-SUMO intermediate. Chemistry & biology.

[127] Lu X, Olsen SK, Capili AD, Cisar JS, Lima CD, Tan DS (2010). Designed semisynthetic protein inhibitors of Ub/Ubl E1 activating enzymes. Journal of the American Chemical Society.

[128] Soucy TA, Smith PG, Milhollen MA, Berger AJ, Gavin JM, Adhikari S, Brownell JE, Burke KE, Cardin DP, Critchley S, Cullis CA, Doucette A, Garnsey JJ, Gaulin JL, Gershman RE, Lublinsky AR (2009). An inhibitor of NEDD8-activating enzyme as a new approach to treat cancer. Nature.

[129] Yao WT, Wu JF, Yu GY, Wang R, Wang K, Li LH, Chen P, Jiang YN, Cheng H, Lee HW, Yu J, Qi H, Yu XJ, Wang P, Chu YW, Yang M (2014). Suppression of tumor angiogenesis by targeting the protein neddylation pathway. Cell death & disease.

[130] Milhollen MA, Traore T, Adams-Duffy J, Thomas MP, Berger AJ, Dang L, Dick LR, Garnsey JJ, Koenig E, Langston SP, Manfredi M, Narayanan U, Rolfe M, Staudt LM, Soucy TA, Yu J (2010). MLN4924, a NEDD8-activating enzyme inhibitor, is active in diffuse large B-cell lymphoma models: rationale for treatment of NF-{kappa}B-dependent lymphoma. Blood.

[131] Ceccarelli DF, Tang X, Pelletier B, Orlicky S, Xie W, Plantevin V, Neculai D, Chou YC, Ogunjimi A, Al-Hakim A, Varelas X, Koszela J, Wasney GA, Vedadi M, Dhe-Paganon S, Cox S (2011). An allosteric inhibitor of the human Cdc34 ubiquitin-conjugating enzyme. Cell.

[132] Pulvino M, Liang Y, Oleksyn D, DeRan M, Van Pelt E, Shapiro J, Sanz I, Chen L, Zhao J (2012). Inhibition of proliferation and survival of diffuse large B-cell lymphoma cells by a small-molecule inhibitor of the ubiquitin-conjugating enzyme Ubc13-Uev1A. Blood.

[133] Varshavsky A (2012). The ubiquitin system, an immense realm. Annual review of biochemistry.

[134] Mattern MR, Wu J, Nicholson B (2012). Ubiquitin-based anticancer therapy: carpet bombing with proteasome inhibitors vs surgical strikes with E1, E2, E3, or DUB inhibitors. Biochimica et biophysica acta.

[135] de Bie P, Ciechanover A (2011). Ubiquitination of E3 ligases: self-regulation of the ubiquitin system via proteolytic and non-proteolytic mechanisms. Cell death and differentiation.

[136] Fan YH, Cheng J, Vasudevan SA, Dou J, Zhang H, Patel RH, Ma IT, Rojas Y, Zhao Y, Yu Y, Zhang H, Shohet JM, Nuchtern JG, Kim ES, Yang J (2013). USP7 inhibitor P22077 inhibits neuroblastoma growth via inducing p53-mediated apoptosis. Cell death & disease.

[137] Mazza D, Infante P, Colicchia V, Greco A, Alfonsi R, Siler M, Antonucci L, Po A, De Smaele E, Ferretti E, Capalbo C, Bellavia D, Canettieri G, Giannini G, Screpanti I, Gulino A (2013). PCAF ubiquitin ligase activity inhibits Hedgehog/Gli1 signaling in p53-dependent response to genotoxic stress. Cell death and differentiation.

[138] Sermeus A, Michiels C (2011). Reciprocal influence of the p53 and the hypoxic pathways. Cell death & disease.

[139] Vousden KH, Prives C (2009). Blinded by the Light: The Growing Complexity of p53. Cell.

[140] Chen YC, Chan JY, Chiu YL, Liu ST, Lozano G, Wang SL, Ho CL, Huang SM (2013). Grail as a molecular determinant for the functions of the tumor suppressor p53 in tumorigenesis. Cell death and differentiation.

[141] Olivier M, Eeles R, Hollstein M, Khan MA, Harris CC, Hainaut P (2002). The IARC TP53 database: new online mutation analysis and recommendations to users. Human mutation.

[142] Oliner JD, Kinzler KW, Meltzer PS, George DL, Vogelstein B (1992). Amplification of a gene encoding a p53-associated protein in human sarcomas. Nature.

[143] Hoe KK, Verma CS, Lane DP (2014). Drugging the p53 pathway: understanding the route to clinical efficacy. Nature reviews Drug discovery.

[144] Ray-Coquard I, Blay JY, Italiano A, Le Cesne A, Penel N, Zhi J, Heil F, Rueger R, Graves B, Ding M, Geho D, Middleton SA, Vassilev LT, Nichols GL, Bui BN (2012). Effect of the MDM2 antagonist RG7112 on the P53 pathway in patients with MDM2-amplified, well-differentiated or dedifferentiated liposarcoma: an exploratory proof-of-mechanism study. The lancet oncology.

[145] Kracikova M, Akiri G, George A, Sachidanandam R, Aaronson SA (2013). A threshold mechanism mediates p53 cell fate decision between growth arrest and apoptosis. Cell death and differentiation.

[146] Chillemi G, Davidovich P, D'Abramo M, Mametnabiev T, Garabadzhiu AV, Desideri A, Melino G (2013). Molecular dynamics of the full-length p53 monomer. Cell cycle (Georgetown, Tex).

[147] Zambetti GP (2014). Expanding the reach of the p53 tumor suppressor network. Cell death and differentiation.

[148] Martynova E, Pozzi S, Basile V, Dolfini D, Zambelli F, Imbriano C, Pavesi G, Mantovani R (2012). Gain-of-function p53 mutants have widespread genomic locations partially overlapping with p63. Oncotarget.

[149] Kadakia MP, De-Fromentel CC, Sabapathy K (2012). The 5th International p63/p73 workshop: much more than just tumour suppression. Cell death and differentiation.

[150] Amelio I, Grespi F, Annicchiarico-Petruzzelli M, Melino G (2012). p63 the guardian of human reproduction. Cell cycle (Georgetown, Tex).

[151] Levine AJ, Tomasini R, McKeon FD, Mak TW, Melino G (2011). The p53 family: guardians of maternal reproduction. Nat Rev Mol Cell Bio.

[152] Tomasini R, Mak TW, Melino G (2008). The impact of p53 and p73 on aneuploidy and cancer. Trends in cell biology.

[153] Billon N, Terrinoni A, Jolicoeur C, McCarthy A, Richardson WD, Melino G, Raff M (2004). Roles for p53 and p73 during oligodendrocyte development. Development.

[154] Masse I, Barbollat-Boutrand L, Molina M, Berthier-Vergnes O, Joly-Tonetti N, Martin MT, Caron de Fromentel C, Kanitakis J, Lamartine J (2012). Functional interplay between p63 and p53 controls RUNX1 function in the transition from proliferation to differentiation in human keratinocytes. Cell death & disease.

[155] Paris M, Rouleau M, Puceat M, Aberdam D (2012). Regulation of skin aging and heart development by TAp63. Cell death and differentiation.

[156] Chari NS, Romano RA, Koster MI, Jaks V, Roop D, Flores ER, Teglund S, Sinha S, Gruber W, Aberger F, Medeiros LJ, Toftgard R, McDonnell TJ (2013). Interaction between the TP63 and SHH pathways is an important determinant of epidermal homeostasis. Cell death and differentiation.

[157] Candi E, Rufini A, Terrinoni A, Giamboi-Miraglia A, Lena AM, Mantovani R, Knight R, Melino G (2007). DeltaNp63 regulates thymic development through enhanced expression of FgfR2 and Jag2. Proceedings of the National Academy of Sciences of the United States of America.

[158] Tomasini R, Secq V, Pouyet L, Thakur AK, Wilhelm M, Nigri J, Vasseur S, Berthezene P, Calvo E, Melino G, Mak TW, Iovanna JL (2013). TAp73 is required for macrophage-mediated innate immunity and the resolution of inflammatory responses. Cell death and differentiation.

[159] Yallowitz AR, Alexandrova EM, Talos F, Xu S, Marchenko ND, Moll UM (2014). p63 is a prosurvival factor in the adult mammary gland during post-lactational involution, affecting PI-MECs and ErbB2 tumorigenesis. Cell death and differentiation.

[160] Burnley P, Rahman M, Wang H, Zhang Z, Sun X, Zhuge Q, Su DM (2013). Role of the p63-FoxN1 regulatory axis in thymic epithelial cell homeostasis during aging. Cell death & disease.

[161] Rufini A, Niklison-Chirou MV, Inoue S, Tomasini R, Harris IS, Marino A, Federici M, Dinsdale D, Knight RA, Melino G, Mak TW (2012). TAp73 depletion accelerates aging through metabolic dysregulation. Gene Dev.

[162] Celardo I, Grespi F, Antonov A, Bernassola F, Garabadgiu AV, Melino G, Amelio I (2013). Caspase-1 is a novel target of p63 in tumor suppression. Cell death & disease.

[163] Manzl C, Fava LL, Krumschnabel G, Peintner L, Tanzer MC, Soratroi C, Bock FJ, Schuler F, Luef B, Geley S, Villunger A (2013). Death of p53-defective cells triggered by forced mitotic entry in the presence of DNA damage is not uniquely dependent on Caspase-2 or the PIDDosome. Cell death & disease.

[164] Melino G, Knight RA, Nicotera P (2005). How many ways to die?. How many different models of cell death? Cell death and differentiation.

[165] Evangelou K, Bartkova J, Kotsinas A, Pateras IS, Liontos M, Velimezi G, Kosar M, Liloglou T, Trougakos IP, Dyrskjot L, Andersen CL, Papaioannou M, Drosos Y, Papafotiou G, Hodny Z, Sosa-Pineda B (2013). The DNA damage checkpoint precedes activation of ARF in response to escalating oncogenic stress during tumorigenesis. Cell death and differentiation.

[166] Louwen F, Yuan JP (2013). Battle of the eternal rivals: restoring functional p53 and inhibiting Polo-like kinase 1 as cancer therapy. Oncotarget.

[167] Shekhar MPV, Kato I, Nangia-Makker P, Tait L (2013). Comedo-DCIS is a precursor lesion for basal-like breast carcinoma: identification of a novel p63/Her2/neu expressing subgroup. Oncotarget.

[168] Giacobbe A, Bongiorno-Borbone L, Bernassola F, Terrinoni A, Markert EK, Levine AJ, Feng Z, Agostini M, Zolla L, Agro AF, Notterman DA, Melino G, Peschiaroli A (2013). p63 regulates glutaminase 2 expression. Cell cycle (Georgetown, Tex).

[169] Montero J, Dutta C, van Bodegom D, Weinstock D, Letai A (2013). p53 regulates a non-apoptotic death induced by ROS. Cell death and differentiation.

[170] He Z, Liu H, Agostini M, Yousefi S, Perren A, Tschan MP, Mak TW, Melino G, Simon HU (2013). p73 regulates autophagy and hepatocellular lipid metabolism through a transcriptional activation of the ATG5 gene. Cell death and differentiation.

[171] Huang YP, Guerrero-Preston R, Ratovitski EA (2012). Phospho-Delta Np63 alpha-dependent regulation of autophagic signaling through transcription and micro-RNA modulation. Cell cycle (Georgetown, Tex).

[172] Selvarajah J, Nathawat K, Moumen A, Ashcroft M, Carroll VA (2013). Chemotherapy-mediated p53-dependent DNA damage response in clear cell renal cell carcinoma: role of the mTORC1/2 and hypoxia-inducible factor pathways. Cell death & disease.

[173] Neise D, Sohn D, Stefanski A, Goto H, Inagaki M, Wesselborg S, Budach W, Stuhler K, Janicke RU (2013). The p90 ribosomal S6 kinase (RSK) inhibitor BI-D1870 prevents gamma irradiation-induced apoptosis and mediates senescence via RSK- and p53-independent accumulation of p21WAF1/CIP1. Cell death & disease.

[174] Huang YP, Jeong JS, Okamura J, Sook-Kim M, Zhu H, Guerrero-Preston R, Ratovitski EA (2012). Global tumor protein p53/p63 interactome Making a case for cisplatin chemoresistance. Cell cycle (Georgetown, Tex).

[175] Huang YP, Kesselman D, Kizub D, Guerrero-Preston R, Ratovitski EA (2013). Phospho-Delta Np63 alpha/microRNA feedback regulation in squamous carcinoma cells upon cisplatin exposure. Cell cycle (Georgetown, Tex).

[176] Mattiske S, Ho K, Noll JE, Neilsen PM, Callen DF, Suetani RJ (2013). TAp63 regulates oncogenic miR-155 to mediate migration and tumour growth. Oncotarget.

[177] Tucci P, Agostini M, Grespi F, Markert EK, Terrinoni A, Vousden KH, Muller PA, Dotsch V, Kehrloesser S, Sayan BS, Giaccone G, Lowe SW, Takahashi N, Vandenabeele P, Knight RA, Levine AJ (2012). Loss of p63 and its microRNA-205 target results in enhanced cell migration and metastasis in prostate cancer. Proceedings of the National Academy of Sciences of the United States of America.

[178] Marcel V, Petit I, Murray-Zmijewski F, Goullet de Rugy T, Fernandes K, Meuray V, Diot A, Lane DP, Aberdam D, Bourdon JC (2012). Diverse p63 and p73 isoforms regulate Delta133p53 expression through modulation of the internal TP53 promoter activity. Cell death and differentiation.

[179] Melino G (2011). p63 is a suppressor of tumorigenesis and metastasis interacting with mutant p53. Cell death and differentiation.

[180] Neilsen PM, Noll JE, Suetani RJ, Schulz RB, Al-Ejeh F, Evdokiou A, Lane DP, Callen DF (2011). Mutant p53 uses p63 as a molecular chaperone to alter gene expression and induce a pro-invasive secretome. Oncotarget.

[181] Luh LM, Kehrloesser S, Deutsch GB, Gebel J, Coutandin D, Schafer B, Agostini M, Melino G, Dotsch V (2013). Analysis of the oligomeric state and transactivation potential of TAp73alpha. Cell death and differentiation.

[182] Yu Y, Huang H, Li J, Zhang J, Gao J, Lu B, Huang C (2013). GADD45 beta mediates p53 protein degradation via Src/PP2A/MDM2 pathway upon arsenite treatment. Cell death & disease.

[183] Melino S, Bellomaria A, Nepravishta R, Paci R, Melino G (2014). p63 threonine phosphorylation signals the interaction with the WW domain of the E3 ligase Itch. Cell cycle (Georgetown, Tex).

[184] Strano S, Munarriz E, Rossi M, Castagnoli L, Shaul Y, Sacchi A, Oren M, Sudol M, Cesareni G, Blandino G (2001). Physical interaction with Yes-associated protein enhances p73 transcriptional activity. Journal of Biological Chemistry.

[185] Levy D, Adamovich Y, Reuven N, Shaul Y (2007). The Yes-associated protein 1 stabilizes p73 by preventing Itch-mediated ubiquitination of p73. Cell death and differentiation.

[186] Danovi SA, Rossi M, Gudmundsdottir K, Yuan M, Melino G, Basu S (2008). Yes-Associated Protein (YAP) is a critical mediator of c-Jun-dependent apoptosis. Cell death and differentiation.

[187] Adamovich Y, Adler J, Meltser V, Reuven N, Shaul Y (2014). AMPK couples p73 with p53 in cell fate decision. Cell death and differentiation.

[188] Oberoi-Khanuja TK, Murali A, Rajalingam K (2013). IAPs on the move: role of inhibitors of apoptosis proteins in cell migration. Cell death & disease.

[189] Tenev T, Ditzel M, Zachariou A, Meier P (2007). The antiapoptotic activity of insect IAPs requires activation by an evolutionarily conserved mechanism. Cell death and differentiation.

[190] Yang WS, Cooke M, Duckett CS, Yang XL, Dorsey JF (2014). Distinctive effects of the cellular inhibitor of apoptosis protein c-IAP2 through stabilization by XIAP in glioblastoma multiforme cells. Cell cycle (Georgetown, Tex).

[191] Dubrez L, Berthelet J, Glorian V (2013). IAP proteins as targets for drug development in oncology. OncoTargets and therapy.

[192] Bertrand MJ, Milutinovic S, Dickson KM, Ho WC, Boudreault A, Durkin J, Gillard JW, Jaquith JB, Morris SJ, Barker PA (2008). cIAP1 and cIAP2 facilitate cancer cell survival by functioning as E3 ligases that promote RIP1 ubiquitination. Molecular cell.

[193] Festjens N, Vanden Berghe T, Cornelis S, Vandenabeele P (2007). RIP1, a kinase on the crossroads of a cell's decision to live or die. Cell death and differentiation.

[194] Wu H, Tschopp J, Lin SC (2007). Smac mimetics and TNFalpha: a dangerous liaison?. Cell.

[195] Tseng PH, Matsuzawa A, Zhang W, Mino T, Vignali DA, Karin M (2010). Different modes of ubiquitination of the adaptor TRAF3 selectively activate the expression of type I interferons and proinflammatory cytokines. Nature immunology.

[196] Wang G, Chan CH, Gao Y, Lin HK (2012). Novel roles of Skp2 E3 ligase in cellular senescence, cancer progression, and metastasis. Chinese journal of cancer.

[197] Bloom J, Pagano M (2003). Deregulated degradation of the cdk inhibitor p27 and malignant transformation. Seminars in cancer biology.

[198] Carrano AC, Eytan E, Hershko A, Pagano M (1999). SKP2 is required for ubiquitin-mediated degradation of the CDK inhibitor p27. Nature cell biology.

[199] Gstaiger M, Jordan R, Lim M, Catzavelos C, Mestan J, Slingerland J, Krek W (2001). Skp2 is oncogenic and overexpressed in human cancers. Proceedings of the National Academy of Sciences of the United States of America.

[200] Tan P, Cady B, Wanner M, Worland P, Cukor B, Magi-Galluzzi C, Lavin P, Draetta G, Pagano M, Loda M (1997). The cell cycle inhibitor p27 is an independent prognostic marker in small (T1a,b) invasive breast carcinomas. Cancer research.

[201] Chan CH, Morrow JK, Li CF, Gao Y, Jin G, Moten A, Stagg LJ, Ladbury JE, Cai Z, Xu D, Logothetis CJ, Hung MC, Zhang S, Lin HK (2013). Pharmacological inactivation of Skp2 SCF ubiquitin ligase restricts cancer stem cell traits and cancer progression. Cell.

[202] Ungermannova D, Lee J, Zhang G, Dallmann HG, McHenry CS, Liu X (2013). High-throughput screening AlphaScreen assay for identification of small-molecule inhibitors of ubiquitin E3 ligase SCFSkp2-Cks1. Journal of biomolecular screening.

[203] Wu TY, Wagner KW, Bursulaya B, Schultz PG, Deveraux QL (2003). Development and characterization of nonpeptidic small molecule inhibitors of the XIAP/caspase-3 interaction. Chemistry & biology.

[204] Aghajan M, Jonai N, Flick K, Fu F, Luo M, Cai X, Ouni I, Pierce N, Tang X, Lomenick B, Damoiseaux R, Hao R, Del Moral PM, Verma R, Li Y, Li C (2010). Chemical genetics screen for enhancers of rapamycin identifies a specific inhibitor of an SCF family E3 ubiquitin ligase. Nature biotechnology.

[205] Orlicky S, Tang X, Neduva V, Elowe N, Brown ED, Sicheri F, Tyers M (2010). An allosteric inhibitor of substrate recognition by the SCF(Cdc4) ubiquitin ligase. Nature biotechnology.

[206] Reyes-Turcu FE, Ventii KH, Wilkinson KD (2009). Regulation and cellular roles of ubiquitin-specific deubiquitinating enzymes. Annual review of biochemistry.

[207] Ernst A, Avvakumov G, Tong J, Fan Y, Zhao Y, Alberts P, Persaud A, Walker JR, Neculai AM, Neculai D, Vorobyov A, Garg P, Beatty L, Chan PK, Juang YC, Landry MC (2013). A strategy for modulation of enzymes in the ubiquitin system. Science (New York, NY).

[208] Zhang S, Du-Cuny L (2009). Development and evaluation of a new statistical model for structure-based high-throughput virtual screening. International journal of bioinformatics research and applications.

[209] Lavecchia A, Di Giovanni C, Cerchia C, Russo A, Russo G, Novellino E (2013). Discovery of a novel small molecule inhibitor targeting the frataxin/ubiquitin interaction via structure-based virtual screening and bioassays. Journal of medicinal chemistry.

[210] Higueruelo AP, Jubb H, Blundell TL (2013). Protein-protein interactions as druggable targets: recent technological advances. Current opinion in pharmacology.

[211] Blackwell HE, Grubbs RH (1989). Highly Efficient Synthesis of Covalently Cross-Linked Peptide Helices by Ring-Closing Metathesis. Angew Chem Int Ed.

[212] Bernal F, Wade M, Godes M, Davis TN, Whitehead DG, Kung AL, Wahl GM, Walensky LD (2010). A stapled p53 helix overcomes HDMX-mediated suppression of p53. Cancer cell.

[213] Brown CJ, Quah ST, Jong J, Goh AM, Chiam PC, Khoo KH, Choong ML, Lee MA, Yurlova L, Zolghadr K, Joseph TL, Verma CS, Lane DP (2013). Stapled peptides with improved potency and specificity that activate p53. ACS chemical biology.

[214] Chang YS, Graves B, Guerlavais V, Tovar C, Packman K, To KH, Olson KA, Kesavan K, Gangurde P, Mukherjee A, Baker T, Darlak K, Elkin C, Filipovic Z, Qureshi FZ, Cai H (2013). Stapled alpha-helical peptide drug development: a potent dual inhibitor of MDM2 and MDMX for p53-dependent cancer therapy. Proceedings of the National Academy of Sciences of the United States of America.

[215] Buckley DL, Gustafson JL, Van Molle I, Roth AG, Tae HS, Gareiss PC, Jorgensen WL, Ciulli A, Crews CM (2012). Small-molecule inhibitors of the interaction between the E3 ligase VHL and HIF1alpha. Angewandte Chemie (International ed in English).

[216] Buckley DL, Van Molle I, Gareiss PC, Tae HS, Michel J, Noblin DJ, Jorgensen WL, Ciulli A, Crews CM (2012). Targeting the von Hippel-Lindau E3 ubiquitin ligase using small molecules to disrupt the VHL/HIF-1alpha interaction. Journal of the American Chemical Society.

[217] Boisclair MD, McClure C, Josiah S, Glass S, Bottomley S, Kamerkar S, Hemmila I (2000). Development of a ubiquitin transfer assay for high throughput screening by fluorescence resonance energy transfer. Journal of biomolecular screening.

[218] Davydov IV, Woods D, Safiran YJ, Oberoi P, Fearnhead HO, Fang S, Jensen JP, Weissman AM, Kenten JH, Vousden KH (2004). Assay for ubiquitin ligase activity: high-throughput screen for inhibitors of HDM2. Journal of biomolecular screening.

[219] Huang J, Sheung J, Dong G, Coquilla C, Daniel-Issakani S, Payan DG (2005). High-throughput screening for inhibitors of the e3 ubiquitin ligase APC. Methods in enzymology.

[220] Kus B, Gajadhar A, Stanger K, Cho R, Sun W, Rouleau N, Lee T, Chan D, Wolting C, Edwards A, Bosse R, Rotin D (2005). A high throughput screen to identify substrates for the ubiquitin ligase Rsp5. The Journal of biological chemistry.

[221] Herman AG, Hayano M, Poyurovsky MV, Shimada K, Skouta R, Prives C, Stockwell BR (2011). Discovery of Mdm2-MdmX E3 ligase inhibitors using a cell-based ubiquitination assay. Cancer discovery.

[222] Scheffner M, Kumar S (2014). Mammalian HECT ubiquitin-protein ligases: biological and pathophysiological aspects. Biochimica et biophysica acta.

[223] Rotblat B, Southwell AL, Ehrnhoefer DE, Skotte NH, Metzler M, Franciosi S, Leprivier G, Somasekharan SP, Barokas A, Deng Y, Tang T, Mathers J, Cetinbas N, Daugaard M, Kwok B, Li L (2014). HACE1 reduces oxidative stress and mutant Huntingtin toxicity by promoting the NRF2 response. Proceedings of the National Academy of Sciences of the United States of America.

[224] Rotin D, Kumar S (2009). Physiological functions of the HECT family of ubiquitin ligases. Nature reviews Molecular cell biology.

[225] Marin I (2010). Animal HECT ubiquitin ligases: evolution and functional implications. BMC evolutionary biology.

[226] Salah Z, Cohen S, Itzhaki E, Aqeilan RI (2013). NEDD4 E3 ligase inhibits the activity of the Hippo pathway by targeting LATS1 for degradation. Cell cycle (Georgetown, Tex).

[227] Salah Z, Melino G, Aqeilan RI (2011). Negative regulation of the Hippo pathway by E3 ubiquitin ligase ITCH is sufficient to promote tumorigenicity. Cancer research.

[228] Layfield R, Lowe J, Bedford L (2005). The ubiquitin-proteasome system and neurodegenerative disorders. Essays in biochemistry.

[229] Rubinsztein DC (2006). The roles of intracellular protein-degradation pathways in neurodegeneration. Nature.

[230] Rossi M, Rotblat B, Ansell K, Amelio I, Caraglia M, Misso G, Bernassola F, Cavasotto CN, Knight RA, Ciechanover A, Melino G (2014). High throughput screening for inhibitors of the HECT ubiquitin E3 ligase ITCH identifies antidepressant drugs as regulators of autophagy. Cell death & disease.

[231] Suryaraja R, Anitha M, Anbarasu K, Kumari G, Mahalingam S (2013). The E3 ubiquitin ligase Itch regulates tumor suppressor protein RASSF5/NORE1 stability in an acetylation-dependent manner. Cell death & disease.

[232] Yamagishi Y, Shoji I, Miyagawa S, Kawakami T, Katoh T, Goto Y, Suga H (2011). Natural product-like macrocyclic N-methyl-peptide inhibitors against a ubiquitin ligase uncovered from a ribosome-expressed de novo library. Chemistry & biology.

[233] Yang D, Li L, Liu H, Wu L, Luo Z, Li H, Zheng S, Gao H, Chu Y, Sun Y, Liu J, Jia L (2013). Induction of autophagy and senescence by knockdown of ROC1 E3 ubiquitin ligase to suppress the growth of liver cancer cells. Cell death and differentiation.

[234] Vassilev LT, Vu BT, Graves B, Carvajal D, Podlaski F, Filipovic Z, Kong N, Kammlott U, Lukacs C, Klein C, Fotouhi N, Liu EA (2004). In vivo activation of the p53 pathway by small-molecule antagonists of MDM2. Science (New York, NY).

[235] Ding K, Lu Y, Nikolovska-Coleska Z, Wang G, Qiu S, Shangary S, Gao W, Qin D, Stuckey J, Krajewski K, Roller PP, Wang S (2006). Structure-based design of spiro-oxindoles as potent, specific small-molecule inhibitors of the MDM2-p53 interaction. Journal of medicinal chemistry.

[236] Shangary S, Qin D, McEachern D, Liu M, Miller RS, Qiu S, Nikolovska-Coleska Z, Ding K, Wang G, Chen J, Bernard D, Zhang J, Lu Y, Gu Q, Shah RB, Pienta KJ (2008). Temporal activation of p53 by a specific MDM2 inhibitor is selectively toxic to tumors and leads to complete tumor growth inhibition. Proceedings of the National Academy of Sciences of the United States of America.

[237] Yang Y, Ludwig RL, Jensen JP, Pierre SA, Medaglia MV, Davydov IV, Safiran YJ, Oberoi P, Kenten JH, Phillips AC, Weissman AM, Vousden KH (2005). Small molecule inhibitors of HDM2 ubiquitin ligase activity stabilize and activate p53 in cells. Cancer cell.

[238] Grasberger BL, Lu T, Schubert C, Parks DJ, Carver TE, Koblish HK, Cummings MD, LaFrance LV, Milkiewicz KL, Calvo RR, Maguire D, Lattanze J, Franks CF, Zhao S, Ramachandren K, Bylebyl GR (2005). Discovery and cocrystal structure of benzodiazepinedione HDM2 antagonists that activate p53 in cells. Journal of medicinal chemistry.

[239] Cai Q, Sun H, Peng Y, Lu J, Nikolovska-Coleska Z, McEachern D, Liu L, Qiu S, Yang CY, Miller R, Yi H, Zhang T, Sun D, Kang S, Guo M, Leopold L (2011). A potent and orally active antagonist (SM-406/AT-406) of multiple inhibitor of apoptosis proteins (IAPs) in clinical development for cancer treatment. Journal of medicinal chemistry.

[240] Flygare JA, Beresini M, Budha N, Chan H, Chan IT, Cheeti S, Cohen F, Deshayes K, Doerner K, Eckhardt SG, Elliott LO, Feng B, Franklin MC, Reisner SF, Gazzard L, Halladay J (2012). Discovery of a potent small-molecule antagonist of inhibitor of apoptosis (IAP) proteins and clinical candidate for the treatment of cancer (GDC-0152). Journal of medicinal chemistry.

[241] Cao XR, Lill NL, Boase N, Shi PP, Croucher DR, Shan H, Qu J, Sweezer EM, Place T, Kirby PA, Daly RJ, Kumar S, Yang B (2008). Nedd4 controls animal growth by regulating IGF-1 signaling. Science signaling.

[242] Nagpal P, Plant PJ, Correa J, Bain A, Takeda M, Kawabe H, Rotin D, Bain JR, Batt JA (2012). The ubiquitin ligase Nedd4-1 participates in denervation-induced skeletal muscle atrophy in mice. PloS one.

[243] Liu PY, Xu N, Malyukova A, Scarlett CJ, Sun YT, Zhang XD, Ling D, Su SP, Nelson C, Chang DK, Koach J, Tee AE, Haber M, Norris MD, Toon C, Rooman I (2013). The histone deacetylase SIRT2 stabilizes Myc oncoproteins. Cell death and differentiation.

[244] Yasuda J, Nakao M, Kawaoka Y, Shida H (2003). Nedd4 regulates egress of Ebola virus-like particles from host cells. Journal of virology.

[245] Timmins J, Schoehn G, Ricard-Blum S, Scianimanico S, Vernet T, Ruigrok RW, Weissenhorn W (2003). Ebola virus matrix protein VP40 interaction with human cellular factors Tsg101 and Nedd4. Journal of molecular biology.

[246] Blot V, Perugi F, Gay B, Prevost MC, Briant L, Tangy F, Abriel H, Staub O, Dokhelar MC, Pique C (2004). Nedd4. 1-mediated ubiquitination and subsequent recruitment of Tsg101 ensure HTLV-1 Gag trafficking towards the multivesicular body pathway prior to virus budding. Journal of cell science.

[247] Kikonyogo A, Bouamr F, Vana ML, Xiang Y, Aiyar A, Carter C, Leis J (2001). Proteins related to the Nedd4 family of ubiquitin protein ligases interact with the L domain of Rous sarcoma virus and are required for gag budding from cells. Proceedings of the National Academy of Sciences of the United States of America.

[248] Yasuda J, Hunter E, Nakao M, Shida H (2002). Functional involvement of a novel Nedd4-like ubiquitin ligase on retrovirus budding. EMBO reports.

[249] Bouamr F, Melillo JA, Wang MQ, Nagashima K, de Los Santos M, Rein A, Goff SP (2003). PPPYVEPTAP motif is the late domain of human T-cell leukemia virus type 1 Gag and mediates its functional interaction with cellular proteins Nedd4 and Tsg101 [corrected]. Journal of virology.

[250] Vana ML, Tang Y, Chen A, Medina G, Carter C, Leis J (2004). Role of Nedd4 and ubiquitination of Rous sarcoma virus Gag in budding of virus-like particles from cells. Journal of virology.

[251] Perry WL, Hustad CM, Swing DA, O'Sullivan TN, Jenkins NA, Copeland NG (1998). The itchy locus encodes a novel ubiquitin protein ligase that is disrupted in a18H mice. Nature genetics.

[252] Lifton RP (1995). Genetic determinants of human hypertension. Proceedings of the National Academy of Sciences of the United States of America.

[253] Shi PP, Cao XR, Sweezer EM, Kinney TS, Williams NR, Husted RF, Nair R, Weiss RM, Williamson RA, Sigmund CD, Snyder PM, Staub O, Stokes JB, Yang B (2008). Salt-sensitive hypertension and cardiac hypertrophy in mice deficient in the ubiquitin ligase Nedd4-2. American journal of physiology Renal physiology.

[254] Zhi X, Chen C (2012). WWP1: a versatile ubiquitin E3 ligase in signaling and diseases. Cellular and molecular life sciences : CMLS.

[255] Rivetti di Val Cervo P, Tucci P, Majid A, Lena AM, Agostini M, Bernardini S, Candi E, Cohen G, Nicotera P, Dyer MJ, Melino G (2009). p73, miR106b, miR34a, and Itch in chronic lymphocytic leukemia. Blood.

[256] Zhang L, Haraguchi S, Koda T, Hashimoto K, Nakagawara A (2011). Muscle atrophy and motor neuron degeneration in human NEDL1 transgenic mice. Journal of biomedicine & biotechnology.

[257] Li Y, Ozaki T, Kikuchi H, Yamamoto H, Ohira M, Nakagawara A (2008). A novel HECT-type E3 ubiquitin protein ligase NEDL1 enhances the p53-mediated apoptotic cell death in its catalytic activity-independent manner. Oncogene.

[258] Shinada K, Tsukiyama T, Sho T, Okumura F, Asaka M, Hatakeyama S (2011). RNF43 interacts with NEDL1 and regulates p53-mediated transcription. Biochem Biophys Res Commun.

[259] Zou W, Chen X, Shim JH, Huang Z, Brady N, Hu D, Drapp R, Sigrist K, Glimcher LH, Jones D (2011). The E3 ubiquitin ligase Wwp2 regulates craniofacial development through mono-ubiquitylation of Goosecoid. Nature cell biology.

[260] Chong-Kopera H, Inoki K, Li Y, Zhu T, Garcia-Gonzalo FR, Rosa JL, Guan KL (2006). TSC1 stabilizes TSC2 by inhibiting the interaction between TSC2 and the HERC1 ubiquitin ligase. The Journal of biological chemistry.

[261] Diouf B, Cheng Q, Krynetskaia NF, Yang W, Cheok M, Pei D, Fan Y, Cheng C, Krynetskiy EY, Geng H, Chen S, Thierfelder WE, Mullighan CG, Downing JR, Hsieh P, Pui CH (2011). Somatic deletions of genes regulating MSH2 protein stability cause DNA mismatch repair deficiency and drug resistance in human leukemia cells. Nature medicine.

[262] Miyazaki K, Fujita T, Ozaki T, Kato C, Kurose Y, Sakamoto M, Kato S, Goto T, Itoyama Y, Aoki M, Nakagawara A (2004). NEDL1, a novel ubiquitin-protein isopeptide ligase for dishevelled-1, targets mutant superoxide dismutase-1. The Journal of biological chemistry.

[263] Bekker-Jensen S, Rendtlew Danielsen J, Fugger K, Gromova I, Nerstedt A, Lukas C, Bartek J, Lukas J, Mailand N (2010). HERC2 coordinates ubiquitin-dependent assembly of DNA repair factors on damaged chromosomes. Nature cell biology.

[264] Danielsen JR, Povlsen LK, Villumsen BH, Streicher W, Nilsson J, Wikstrom M, Bekker-Jensen S, Mailand N (2012). DNA damage-inducible SUMOylation of HERC2 promotes RNF8 binding via a novel SUMO-binding Zinc finger. The Journal of cell biology.

[265] Puffenberger EG, Jinks RN, Wang H, Xin B, Fiorentini C, Sherman EA, Degrazio D, Shaw C, Sougnez C, Cibulskis K, Gabriel S, Kelley RI, Morton DH, Strauss KA (2012). A homozygous missense mutation in HERC2 associated with global developmental delay and autism spectrum disorder. Human mutation.

[266] Harlalka GV, Baple EL, Cross H, Kuhnle S, Cubillos-Rojas M, Matentzoglu K, Patton MA, Wagner K, Coblentz R, Ford DL, Mackay DJ, Chioza BA, Scheffner M, Rosa JL, Crosby AH (2013). Mutation of HERC2 causes developmental delay with Angelman-like features. Journal of medical genetics.

[267] Kishino T, Lalande M, Wagstaff J (1997). UBE3A/E6-AP mutations cause Angelman syndrome. Nature genetics.

[268] Matsuura T, Sutcliffe JS, Fang P, Galjaard RJ, Jiang YH, Benton CS, Rommens JM, Beaudet AL (1997). De novo truncating mutations in E6-AP ubiquitin-protein ligase gene (UBE3A) in Angelman syndrome. Nature genetics.

[269] Glessner JT, Wang K, Cai G, Korvatska O, Kim CE, Wood S, Zhang H, Estes A, Brune CW, Bradfield JP, Imielinski M, Frackelton EC, Reichert J, Crawford EL, Munson J, Sleiman PM (2009). Autism genome-wide copy number variation reveals ubiquitin and neuronal genes. Nature.

[270] Hogart A, Wu D, LaSalle JM, Schanen NC (2010). The comorbidity of autism with the genomic disorders of chromosome 15q11. 2-q13. Neurobiology of disease.

[271] Durfee LA, Lyon N, Seo K, Huibregtse JM (2010). The ISG15 conjugation system broadly targets newly synthesized proteins: implications for the antiviral function of ISG15. Molecular cell.

[272] Zhao C, Hsiang TY, Kuo RL, Krug RM (2010). ISG15 conjugation system targets the viral NS1 protein in influenza A virus-infected cells. Proceedings of the National Academy of Sciences of the United States of America.

[273] Adhikary S, Marinoni F, Hock A, Hulleman E, Popov N, Beier R, Bernard S, Quarto M, Capra M, Goettig S, Kogel U, Scheffner M, Helin K, Eilers M (2005). The ubiquitin ligase HectH9 regulates transcriptional activation by Myc and is essential for tumor cell proliferation. Cell.

[274] Chen D, Brooks CL, Gu W (2006). ARF-BP1 as a potential therapeutic target. British journal of cancer.

[275] Confalonieri S, Quarto M, Goisis G, Nuciforo P, Donzelli M, Jodice G, Pelosi G, Viale G, Pece S, Di Fiore PP (2009). Alterations of ubiquitin ligases in human cancer and their association with the natural history of the tumor. Oncogene.

[276] Beaudenon S, Huibregtse JM (2008). HPV E6, E6AP and cervical cancer. BMC biochemistry.

[277] Clancy JL, Henderson MJ, Russell AJ, Anderson DW, Bova RJ, Campbell IG, Choong DY, Macdonald GA, Mann GJ, Nolan T, Brady G, Olopade OI, Woollatt E, Davies MJ, Segara D, Hacker NF (2003). EDD, the human orthologue of the hyperplastic discs tumour suppressor gene, is amplified and overexpressed in cancer. Oncogene.

[278] Fuja TJ, Lin F, Osann KE, Bryant PJ (2004). Somatic mutations and altered expression of the candidate tumor suppressors CSNK1 epsilon, DLG1, and EDD/hHYD in mammary ductal carcinoma. Cancer research.

[279] Chen D, Yoon JB, Gu W (2010). Reactivating the ARF-p53 axis in AML cells by targeting ULF. Cell cycle (Georgetown, Tex).

[280] Zhang L, Anglesio MS, O'Sullivan M, Zhang F, Yang G, Sarao R, Mai PN, Cronin S, Hara H, Melnyk N, Li L, Wada T, Liu PP, Farrar J, Arceci RJ, Sorensen PH (2007). The E3 ligase HACE1 is a critical chromosome 6q21 tumor suppressor involved in multiple cancers. Nature medicine.

[281] Slade I, Stephens P, Douglas J, Barker K, Stebbings L, Abbaszadeh F, Pritchard-Jones K, Cole R, Pizer B, Stiller C, Vujanic G, Scott RH, Stratton MR, Rahman N (2010). Constitutional translocation breakpoint mapping by genome-wide paired-end sequencing identifies HACE1 as a putative Wilms tumour susceptibility gene.. Journal of medical genetics.

